# Crystal Structures and Luminescence Properties of the Oxonitridolithosilicates *RE*_2_[LiSi_3_N_5_O_2_] (*RE* = Ce, Pr, Nd, Sm, Gd, Tb)

**DOI:** 10.1002/open.70329

**Published:** 2026-11-08

**Authors:** Mike N. van Os, Kilian M. Rießbeck, Markus Seibald, Christiane Stoll, Hubert Huppertz

**Affiliations:** ^1^ Department of General, Inorganic and Theoretical Chemistry University of Innsbruck Innsbruck Austria; ^2^ ams‐OSRAM International GmbH Schwabmünchen Germany

**Keywords:** crystal structure, high‐temperature synthesis, luminescent material, mixed anions, rare‐earth oxonitridolithosilicate, solid‐state reaction

## Abstract

The rare‐earth oxonitridolithosilicate series *RE*_2_[LiSi_3_N_5_O_2_] (*RE* = Ce, Pr, Nd, Sm, Tb) is reported as isotypic representatives of the previously discovered compound Gd_2_[LiSi_3_N_5_O_2_], accessed via high‐temperature solid‐state synthesis. All compounds crystallize in the non‐centrosymmetric tetragonal space group $P\bar{4}2_{1}m$ (no. 113), as determined by single‐crystal X‐ray diffraction and confirmed by Rietveld refinement. The crystal structure is related to the melilite structure type and is built up of a tecto‐lithosilicate comprising corner‐ and edge‐sharing [LiN_4_]‐, [SiN_4_]‐, and [SiN_3_O]‐tetrahedra, with the rare‐earth ions occupying channels within the resulting network. The lanthanoid contraction‐induced decrease of all unit cell parameters across the series is consistent with the structural analysis and is further supported by thermal measurements. These trends indicate a hierarchical distribution of rigidity and flexibility within the framework, thereby establishing this compound class as a model system for structure–property relations in rare‐earth oxonitridolithosilicates. On the rare‐earth site, the intra‐configurational 4f–4f transitions of Tb^3+^ and Nd^3+^ are identified. For Eu^2+^‐activated Tb_2_[LiSi_3_N_5_O_2_], an orange 5d → 4f emission at *λ*_max_ = 618 nm is observed, establishing the potential of this compound class for solid‐state lighting applications.

## Graphical abstract

The first series of rare‐earth oxonitridolithosilicates *RE*_2_[LiSi_3_N_5_O_2_] (*RE* = Ce, Pr, Nd, Sm, Gd, Tb) represents a new isotypic compound class featuring a mixed [LiN_4_]^‐^, [SiN_4_]^‐^, and [SiN_3_O]^−^tetrahedra network. The *RE* ^3+^ site with an oxygen and nitrogen coordination environment governs characteristic 4f → 4f emissions of Tb^3+^ and Nd^3+^, while Eu^2+^ activation of the Tb compound yields orange 5d → 4f emission at *λ*_max_ = 618 nm (2.01 eV), demonstrating the potential of this compound class for solid‐state lighting.

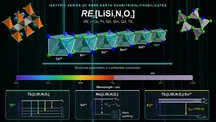

## 1 Introduction

Oxolithosilicates are compounds in which silicon and lithium are tetrahedrally coordinated by oxygen atoms, thereby Li^+^ is not just a charge‐balancing cation but also a structure‐forming component of the anionic substructure. This structural principle was systematically investigated and described by Hoppe and Hofmann in 1988, using the alkali oxolithosilicate K_3_[LiSiO_4_] as a representative example. In the sum formula notation introduced therein, ions belonging to the tetrahedral substructure are enclosed in square brackets, while the remaining cations and anions are placed outside [1]. Concurrent with this work, a series of further alkali oxolithosilicates were reported, including Rb(Li,Na)[Li_3_SiO_4_]_2_ and Rb_2_[Li_2_SiO_4_], whose anionic substructure consists of interconnected [LiO_4_]‐ and [SiO_4_]‐tetrahedra with higher‐coordinated cations embedded within the resulting voids [2, 3].

The structural diversity within this compound class reflects a broad range of accessible structure motifs. A particularly significant modification is achieved through the partial substitution of oxygen by nitrogen within the lithosilicate substructure, leading to the formation of oxonitridolithosilicates. Due to the higher charge and increased covalent character of nitrogen compared with oxygen, this substitution can enhance the connectivity of the tetrahedral network, thereby influencing the framework rigidity and enabling a systematic tuning of the structural, thermal, and optical properties [4, 5, 6, 7, 8]. Therefore, luminescent compounds for phosphor‐converted light‐emitting diodes (pc‐LEDs) are a central research field in this class of materials. Effective phosphors must combine high thermal and chemical stability with resistance to thermal quenching and high photoluminescence quantum yield (PLQY). An important role in this context is the activation of (litho)silicates with Eu^2+^, as its mostly electric‐dipole allowed 4f^6^5d^1 ^→ 4f^7^ transitions provide high absorption and emission probabilities. In many (litho)silicates, the combination of a suitable bandgap and the crystal‐field‐dependent position of the Eu^2+^ 5d levels results in efficient excitation by blue or near‐ultraviolet LEDs (near‐UV LEDs). The resulting emission, typically spanning the blue to red spectral region, is highly sensitive to the local crystal‐field strength, enabling systematic tuning of the emission color [9, 10, 11]. A well‐known example is the (Ca,Sr,Ba)[Si_2_O_2_N_2_]:Eu^2+^ system, which combines thermal and chemical stability, exhibiting emission maxima ranging from 494 to 570 nm (2.18 – 2.51 eV) [12, 13]. Similarly, Rb(Li,Na)[Li_3_SiO_4_]_2_:Eu^2+^ has a narrow‐band emission at 530 and 523 nm with a full width at half maximum (*fwhm*) of approximately 42 and 41 nm (0.19 eV, 1501 cm^−1^), showing the potential of lithosilicate hosts for tailored luminescence [14]. Further Eu^2+^‐activated systems, such as A_4_[Li_3_SiO_4_]_4_ (A = Na, K, Rb, and Cs), (Ca,Sr,Ba)_2_[Si_5_N_8_], or SrAl_2–*x*_Li_2+*x*_O_2+2*x*_N_2–2*x*_, have demonstrated the capacity to adjust correlated color temperatures (CCTs) in phosphor‐converted light‐emitting diodes (pc‐LEDs) [15, 16, 17, 18, 19]. The range of Eu^2+^‐activated materials was further extended by narrow‐band emitters of technological relevance. Sr[Li_2_Al_2_O_2_N_2_]:Eu^2+^ exhibits a narrow‐band red emission at 614 nm (2.02 eV) with a *fwhm* of 48 nm (0.16 eV, 1180 cm^−1^), making it a promising candidate for high color rendering applications [20, 21]. Another efficient narrow‐band red‐emitting phosphor is (Ca,Sr)[LiAl_3_N_4_]:Eu^2+^ (CLA, SLA) with an exceptionally small *fwhm* (0.15, 0.17 eV), making it highly suitable for backlighting applications in liquid crystal displays (LCDs) [7, 22]. Ba_3_[Si_6_O_12_N_2_]:Eu^2+^, on the other hand, contributes to the green spectral region with good thermal stability and high quantum efficiency, further demonstrating the spectral versatility achievable within oxonitridosilicates and related host structures [23, 24, 25].

The incorporation of rare‐earth cations into lithosilicate frameworks further extends their scientific and technological significance, particularly by enabling the development of functional materials with luminescence properties. Our group provided the first insights into the emission properties of a rare‐earth oxonitridolithosilicate, reporting on Eu^2+^‐ and Ce^3+^‐activated Gd_2_[LiSi_3_N_5_O_2_] and demonstrating with Li_8_ *RE*[LiSi_4_N_4_O_8_] (*RE* = Y, La, Gd, Dy, Yb, Lu) that lithium rare‐earth oxonitridolithosilicates are capable of yielding phosphor performance comparable to or even exceeding in some properties that of the benchmark material YAG:Ce^3+^ for white LEDs (wLEDs) [26, 27]. Related lithium (litho)silicates, such as the first discovered lithium rare‐earth oxonitridosilicate LiLa_5_Si_4_N_10_O in 2012 or Li[Li_2_SiNO_2_], further illustrate the range of accessible structural motifs [28, 29].

Beyond the Eu^2+^‐activated systems discussed above, Tb^3+^ and Nd^3+^ represent luminescent centers of considerable independent interest. The technological relevance of Tb^3+^ luminescence is well established in green‐emitting phosphors, like the compounds LaPO_4_:Ce^3+^,Tb^3+^ (LAP) and CeMgAl_11_O_19_:Tb^3+^ (CAT) and have served as the green component in tricolor fluorescent discharge lamps since the 1970s, where efficient Ce^3+ ^→ Tb^3+^ energy transfer enabled the utilization of the mercury 254 nm line [30]. Nd^3+^‐activated inorganic phosphors like Bi_2_SiO_5_, Sr_6_Y(PO_4_)_5_, or LaPO_4_ generate characteristic near‐infrared (NIR) emission bands from 700 to 1350 nm, which fall within the first and second biological transparency windows. Additionally, these transitions are particularly well suited for optical sensing applications, enabling noncontact luminescence thermometry [31, 32, 33].

Building upon the work of our group on Gd_2_[LiSi_3_N_5_O_2_], the present study extends the structural and luminescence analysis to the isotypic series *RE*_2_[LiSi_3_N_5_O_2_] (*RE* = Ce, Pr, Nd, Sm, Tb) [27]. Beyond Eu^2+^ activation, the emission characteristics from the rare‐earth constituents themselves are investigated, with the aim of establishing structure–property relations within this emerging compound class.

## 2 Results and Discussion

### 2.1 Crystal Structure

Single‐crystal X‐ray diffraction data proved that the phases *RE*_2_[LiSi_3_N_5_O_2_] (*RE* =Pr, Nd, Tb) crystallize isotypically to Gd_2_[LiSi_3_N_5_O_2_] [27]. The corresponding measurement and refinement details are listed in Table 1. The Wyckoff‐positions, atomic coordinates, and equivalent displacement parameters for Nd_2_[LiSi_3_N_5_O_2_] are noted in Table 2 as an example. Further information on the other compounds about the crystallographic data and structure refinements, Wyckoff‐positions, atomic coordinates, equivalent and anisotropic displacement parameters can be found in Tables S1–S7. The compounds *RE*_2_[LiSi_3_N_5_O_2_] (*RE* = Ce, Sm) were analyzed by Rietveld refinement, and they crystallize also isotypically to Gd_2_[LiSi_3_N_5_O_2_] [27]. The corresponding Rietveld refinement of Sm_2_[LiSi_3_N_5_O_2_] is shown as an example in Figure 1. The compound Sm_2_[LiSi_3_N_5_O_2_] is identified to represent the main phase of the reaction product (proportion of 61 wt%). Further Rietveld refinements for the compounds *RE*_2_[LiSi_3_N_5_O_2_] (*RE *= Ce, Pr, Nd, Gd, Tb) were conducted, and they are illustrated in Figures S1–S3 with further parameters listed in Tables S8–S10.

**TABLE 1:** Crystallographic data and structure refinements of *RE*_2_[LiSi_3_N_5_O_2_] (*RE* = Pr, Nd, Tb) and Gd_2_[LiSi_3_N_5_O_2_]:Ce^3+^ [27].

Parameter	Value			
Empirical formula	Li*RE*_2_Si_3_N_5_O_2_			
*RE*	Pr	Nd	Gd [27]	Tb
Molecular mass/g mol^−1^	475.08	481.74	507.76	511.10
Crystal system	tetragonal			
Space group	$P\bar{4}2_{1}m$ (no. 113)			
Single‐crystal diffractometer	Bruker D8 QUEST PHOTON III C14			
Radiation	Mo K‐L_2,3_ (*λ* = 0.71073 Å)			Cu K‐L_2,3_ (*λ* = 1.54178 Å)
*a*/Å	7.8272(1)	7.7998(1)	7.7178(1)	7.6915(1)
*c*/Å	5.0068(1)	4.9909(2)	4.9324(1)	4.9301(1)
*V*/Å^3^	306.74(2)	303.63(2)	293.80(2)	291.66(2)
*Z*	2			
Calculated density/g cm^−3^	5.144	5.269	5.740	5.820
Temperature/K	293	293	301	296
Reflections total/independent	17 498/1166	7341/797	9254/1045	877/148
*R*_int_	0.0306	0.0274	0.0580	0.0973
Reflections [*I* ≥ 2*σ*(*I*)]	1118	780	991	121
*R*_σ_	0.0123	0.0157	0.0157	0.0740
Data/ref. parameters	1166/38	797/38	1045/36	148/28
Absorption correction	multi‐scan (SADABS‐2016/2) [34]			
Goodness of fit on *F* ^2^	1.130	1.125	1.019	1.233
Flack parameter	0.22(3)	0.25(2)	0.00(2)	0.1710(2)
Final *R*_1_/*wR*_2_ [*I* ≥ 2*σ*(*I*)]	0.0154/0.0363	0.0117/0.0268	0.0184/0.0425	0.0727/0.1340
Final *R*_1_/*wR*_2_ (all data)	0.0171/0.0373	0.0121/0.0270	0.0200/0.0432	0.0956/0.1456
Largest diff. peak/hole/e·Å^−3^	1.384/−1.420	0.750/−0.841	2.201/−1.106	0.518/−0.458

**TABLE 2:** Wyckoff‐positions, atomic coordinates, and equivalent displacement parameters for Nd_2_[LiSi_3_N_5_O_2_] (standard deviations in parentheses).

Atom	Wyckoff‐position	***x***	***y***	***z***	***U***_**eq**_/**Å** ^**2**^
Nd1	4*e*	0.16039(2)	*x* + ½	0.50242(4)	0.00804(5)
Si1	4*e*	0.64379(9)	*x* + ½	0.9394(2)	0.0061(2)
Si2	2*a*	0	0	0	0.0049(2)
O1	4*e*	0.6426(3)	*x* + ½	0.2730(5)	0.0095(4)
N1	8*f*	0.0804(3)	0.1610(3)	0.2058(4)	0.0074(3)
N2	2*c*	0	½	0.1766(8)	0.0063(6)
Li1	2*b*	0	0	½	0.021(2)

**FIGURE 1:**
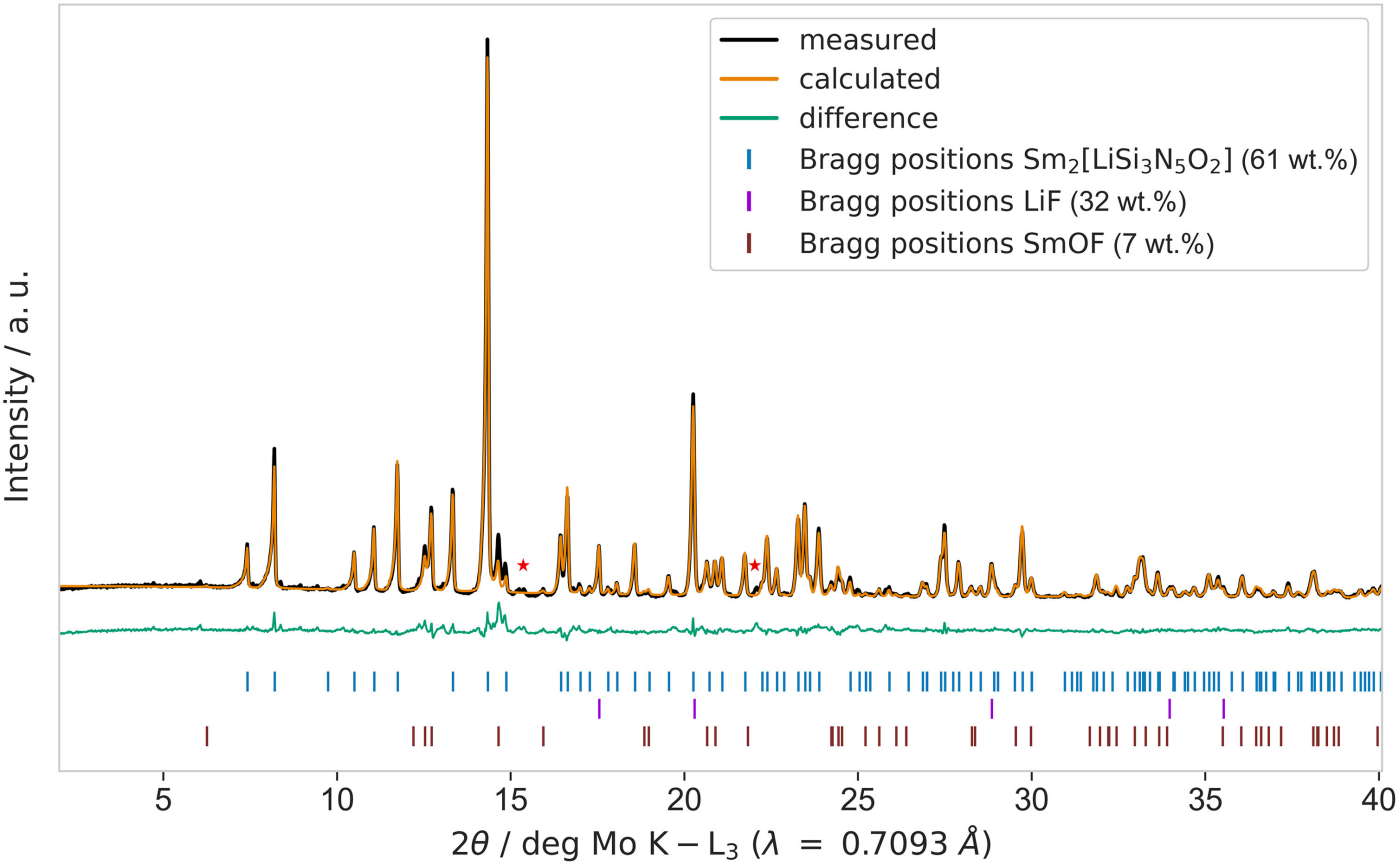
Rietveld fit of the measured powder diffraction data (black line) of Sm_2_[LiSi_3_N_5_O_2_], the calculated pattern based on the theoretical reflections of the phases (orange line), and the difference plot (green line). Tick marks represent the theoretical reflection position of Sm_2_[LiSi_3_N_5_O_2_] (blue tick marks; 61 wt%), LiF (purple tick marks; 32 wt%) and SmOF (brown tick marks; 7 wt%). Reflections that are not identified are asterisked (red).

The Rietveld refinements show that none of the powder samples is phase‐pure. Besides the target phase *RE*_2_[LiSi_3_N_5_O_2_], secondary phases are present in considerable amounts. Furthermore, the refined weight fractions do not represent absolute values. Some reflections cannot be assigned to any known phase (marked with asterisks). As the weight fractions are normalized to the sum of the refined crystalline phases, the contributions of these unidentified and amorphous constituents are not included and the fractions of all refined phases are overestimated accordingly. In addition, the fraction of the weakly scattering LiF next to the strongly scattering rare‐earth phases is likely overestimated due to microabsorption. The values given in the Supporting Information are therefore regarded as semiquantitative and describe only the relative proportions of the identified phases. The title compounds are obtained by a metathesis reaction between *RE*F_3_ and Li_3_N, in which the formation of LiF provides the thermodynamic driving force. LiF is therefore a stoichiometric byproduct and not an avoidable impurity. In addition, fluorides and probably also the forming LiF (*T*_m_ = 845 °C) act as a flux at the reaction temperature and promote the growth of single crystals being essential for the formation of the target phases. LiF fractions above the expected value, as observed for the Ce sample, indicate an incomplete conversion to the target phase. Further side phases, like *RE*OF, form by the reaction of fluoride with oxygen containing species. As they withdraw *RE* cations and oxygen from the reaction mixture, the overall composition is shifted. The secondary phases do not affect the structure determination, which is based on single‐crystal data, but they limit the significance of all measurements on bulk samples. LiF is optically inert in the visible spectral region, whereas the oxide fluorides and oxides may host the activator ions. For this reason, the later discussed measurements were recorded on single crystals or single grains of the target compound.

Single‐crystal X‐ray diffraction data were further evaluated by calculating bond‐valence sums for all crystallographic sites in Nd_2_[LiSi_3_N_5_O_2_] using the bond‐length/bond‐strength (BLBS) and charge distribution (CHARDI) approaches. The resulting values are in good agreement with the expected formal oxidation states (+1 for Li; +4 for Si; +3 for *RE*; −3 for N; −2 for O), thereby confirming the internal charge balance and the reliability of the structural model [35, 36, 37]. The results of the calculations are shown in Table 3. Further calculations for the compounds *RE*_2_[LiSi_3_N_5_O_2_] (*RE* = Pr, Tb) were conducted and the results are listed in Tables S11–S12.

**TABLE 3:** Calculated bond valences using BLBS (∑*V*) and CHARDI‐2015 (∑*Q*) for Nd_2_[LiSi_3_N_5_O_2_] based on single‐crystal data [35, 36, 37].

Atom	Nd1	Li1	Si1	Si2	N1	N2	O1
∑*V*	2.82	0.99	3.86	3.84	−2.79	−3.24	−1.85
∑*Q*	2.99	1.02	3.83	4.08	−2.96	−3.01	−1.99

Furthermore, Madelung Part of Lattice Energy (MAPLE) calculations of the lattice energy were performed to determine the electrostatic consistency of the crystal structures [38, 39]. The results are listed in Table 4. Therefore, electrostatic interactions, which depend on the charge, distance, and the coordination spheres of the ions, were considered. These calculations are particularly helpful to support the single‐crystal X‐ray diffraction data and prove the positions of oxygen and nitrogen as these atoms have nearly identical scattering factors. The partial MAPLE values of all individual atoms are in good agreement with reference values. By comparing MAPLE sums of different nitrides and oxides with the MAPLE values of Nd_2_[LiSi_3_N_5_O_2_], the electrostatic consistency of the refined crystal structures was verified. The MAPLE values were evaluated according to the following reaction equation:

**TABLE 4:** Partial MAPLE values and MAPLE sums in kJ mol^−1^ for Nd_2_[LiSi_3_N_5_O_2_] based on single‐crystal data [38, 39, 40, 41, 42].

	Nd_2_[LiSi_3_N_5_O_2_]	Reference [40, 41, 42]	Compound	MAPLE value (× Factor)
Nd1^3+^	4236.20	3500–5100	Li_3_N [43]	6327.43 (×1/3)
Li1^+^	829.28	550–860	NdN [44]	8501.02 (×2/3)
Si1^4+^	9753.44	9000–10,200	Si_3_N_4_ [45]	53,365.66 (×1)
Si2^4+^	10,000.32	9000–10,200	Nd_2_O_3_ [46]	14,485.12 (×2/3)
N1^3−^	5364.74	4300–6200		
N2^3−^	5650.59	4300–6200		
O1^2−^	2431.78	2000–2800		
∑	70,781.98			70,798.77
Δ	0.02%

(1) $$⅓\;{\mathrm{Li}}_{3}N+⅔\;\text{NdN}+{\mathrm{Si}}_{3}N_{4}+\text{⅔}{\mathrm{Nd}}_{2}O_{3}\to \text{LiN}d_{2}{\mathrm{Si}}_{3}N_{5}O_{2}$$

Further MAPLE values for the compounds *RE*_2_[LiSi_3_N_5_O_2_] (*RE* = Pr, Gd, Tb) were obtained and they are listed in Table S13.

The crystal structure of *RE*_2_[LiSi_3_N_5_O_2_] is most effectively explained by starting from the anionic substructures and subsequently building up the coordination environment of the rare‐earth cation. In the following, the compound Nd_2_[LiSi_3_N_5_O_2_] is used as an example. Our group explained already that Gd_2_[LiSi_3_N_5_O_2_] represents a tecto‐lithosilicate and is a homeotypic derivative of the melilite structure type, particularly åkermanite. According to the Liebau classification, the anionic substructure consists of five corner‐sharing [SiN_4_]‐ and [SiN_3_O]‐tetrahedra forming *fünfer*‐rings and building up layers parallel to the *a–b*‐plane. These layers can be classified as a phyllosilicate. In contrast to åkermanite, additional [LiN_4_]‐tetrahedra are incorporated in Gd_2_[LiSi_3_N_5_O_2_]. These units act as network formers connecting adjacent silicate layers via edge‐sharing tetrahedra, transforming the formally phyllosilicate into a tecto‐lithosilicate [5, 27].

The Nd^3+^ cations occupy the Wyckoff‐position 4*e* in the non‐centrosymmetric space group $P\bar{4}2_{1}m$ (no. 113) and they are coordinated by eight anions forming a distorted squared antiprism, as illustrated in Figure 2a. The coordination sphere consists of five nitrogen and three oxygen atoms with Nd–N distances ranging from 2.401(3) to 2.767(2) Å and Nd–O distances between 2.457(3) and 2.619(2) Å. The broad distribution of bond lengths indicates a significantly distorted coordination polyhedron (Table 5). Building on this coordination environment, the structure can formally be described as an alternating stacking of two distinct layers in an AB sequence shown in Figure 2b. Layer A in Figure 2c consists of tetrahedrally coordinated lithium units ([LiN_4_]) and the Nd^3+^ cations, whereas layer B is formed by a silicate substructure composed of [SiN_4_]‐ and [SiN_3_O]‐tetrahedra. The silicate layer extends in the *a–b*‐plane and is constructed from corner‐sharing tetrahedra that form the mentioned *fünfer*‐rings shown in Figure 2d. These rings are exclusively linked via μ_2_‐N atoms (N^[2]^), which act as bridging ligands between adjacent tetrahedra. Oxygen atoms are incorporated within the [SiN_3_O]‐tetrahedra and are also coordinated to Nd, but they do not contribute to the linkage between the tetrahedral units. Consequently, the connectivity of the substructure is governed by nitrogen anions, whereas the oxygen anions modify the local coordination environment without extending the network.

**FIGURE 2:**
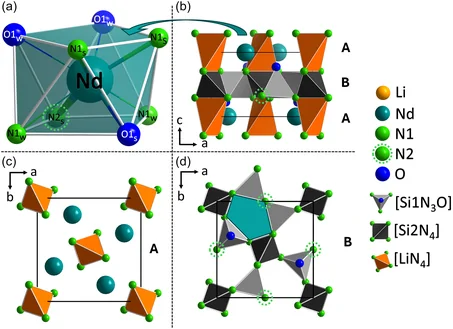
(a) Distorted square antiprism [NdN_5_O_3_], (b) the crystal structure of Nd_2_[LiSi_3_N_5_O_2_] along the crystallographic *b*‐axis, (c) layer A of tetrahedrally coordinated lithium units ([LiN_4_]) and the Nd^3+^ cations, (d) layer B of the silicate substructure composed of [Si1N_3_O]‐ and [Si2N_4_]‐tetrahedra. Additionally, stronger bonds in the distorted square antiprism are marked with thicker bindings in (a) and the void within one of the *fünfer‐*rings is highlighted by a teal pentagon in (d). The tetrahedra are color‐coded according to the central atoms.

**TABLE 5:** Selected bond lengths of the distorted square antiprism [NdN_5_O_3_] in Nd_2_[LiSi_3_N_5_O_2_] (standard deviations in parentheses).

Atoms	Nd–O1_w_	Nd–O1_s_	Nd–N1_s_	Nd–N1_w_	Nd–N2_s_
Bond length/Å	2.619(2)	2.457(3)	2.492(2)	2.767(2)	2.401(3)

Layer A is the lithium‐containing plane and built up from [LiN_4_]‐tetrahedra, which are edge‐linked to the [SiN_4_]‐tetrahedra (Figure 2b). Here, the nitrogen atoms again act as μ_2_‐bridging ligands between Li and Si centers, leading to a mixed μ_2_‐N(Si,Li) connectivity. The interatomic distances for Nd_2_[LiSi_3_N_5_O_2_] are listed in Table S14.

A quantitative description of the tetrahedra distortions within this substructure is obtained using the tetrahedra angle variance *σ* ^2^, defined as

(2) $$\sigma^{2}=\frac{1}{n-1}\sum_{i=1}^{n}{\left(\theta_{i}-109.47^{{}^{\circ}}\right)}^{2}$$

with *n *= 6 for a tetrahedron. This parameter, originally introduced by Robinson et al., allows a direct comparison of deviations from the ideal tetrahedral geometry [47]. The calculated *σ* ^2^ values reveal a pronounced hierarchy of distortions within the structure. The [SiN_4_]‐tetrahedra exhibit a very low value (*σ* ^2^ ≈ 2.06 deg^2^) and are therefore close to ideal geometry, consistent with their role as rigid structure‐forming units. In contrast, the [SiN_3_O]‐tetrahedra show a higher distortion (*σ* ^2^ ≈ 21.3 deg^2^), which can be attributed to the mixed anionic coordination environment, where differences in bond strength between Si–N and Si–O induce angular asymmetry. The [LiN_4_]‐tetrahedron has two angles of 87.4(2)° and four of 121.50(7)° showing extremely large deviations (*σ* ^2^ ≈ 310 deg^2^) and reflecting their weakly bound and highly flexible coordination environment. The interatomic angles and mean values are listed in Table 6.

**TABLE 6:** Selected interatomic angles and mean values (degrees, mentioned in bold) in Nd_2_[LiSi_3_N_5_O_2_] (standard deviations in parentheses).

Atoms	Angle/°	Count	Average value	Angle/°
O1–**Si1**–N2	109.6(2)			
O1–**Si1**–N1	114.6(1)	2×	**Ø O–Si1–N**	112.9(2)
N2–**Si1**–N1	104.3(1)	2×		
N1–**Si1**–N1	108.5(2)		**Ø N–Si1** **–** **N**	105.7(2)
N1–**Si2**–N1	110.4(1)	2×		
N1–**Si2**–N1	107.6(2)	2×		
N1–**Si2**–N1	110.4(1)	2×	**Ø N–Si2–N**	109.5(2)
N1–**Li1**–N1	87.4(2)	2×		
N1–**Li1**–N1	121.50(7)	2×	**Ø N–** **Li1** **–N**	110.1(2)

The connectivity of the tetrahedra via N^[2]^ corners lead to significant deviations of the Si–N–Si angles from linearity (180°), which relates to a maximum relaxation of the anionic substructure, with values of approximately 118.8(2)° for the mixed linkage 2(Si2–N1^[2]^–Si1) and 139.67(4)° for the symmetric linkage (Si1–N2^[2]^–Si1). These angles are shown in Figure 3 and provide a direct measure of tetrahedral tilting within the substructure. A clear correlation between local distortion and network tilting is observed. Nearly ideal [Si2N_4_]‐tetrahedra, which cannot accommodate strain internally, enforce stronger tilting of the silicate substructure, resulting in smaller Si–N–Si angles. In contrast, the more distorted [Si1N_3_O]‐tetrahedra seem to damp part of the structural strain locally, leading to less pronounced tilting and larger Si–N–Si angles. Taking this together and considering the highly flexible edge‐sharing [LiN_4_]‐tetrahedra connected to the [SiN_4_]‐tetrahedra, it results in a direction‐dependent connectivity. The higher in‐plane interconnection of the silicate substructure leads to a more rigid and less flexible network within the *a–b*‐plane, thereby restricting atomic motion more strongly compared to the *c* direction. This interplay is essential for stabilizing the observed topology and is consistent with structural principles observed in related (oxonitrido)lithosilicates and melilite‐type compounds [42, 48, 49, 50, 51].

**FIGURE 3:**
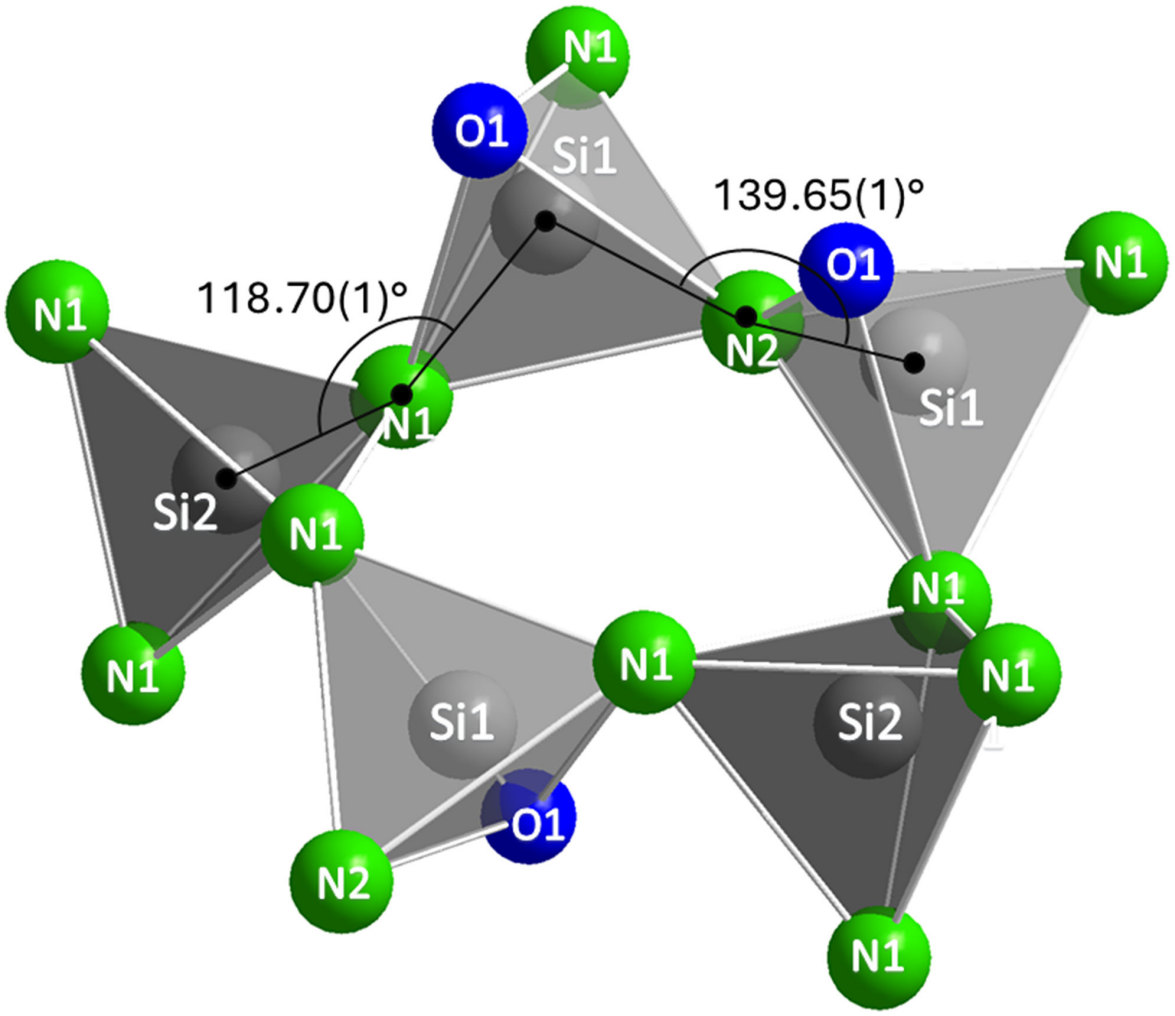
Illustration of the *fünfer*‐rings in the anionic silicate substructure, showing the connectivity of the tetrahedra via nitrogen atoms and the corresponding linkage angles.

### 2.2 Temperature‐Dependent Powder X‐ray Diffraction

The direction‐dependent connectivity of the network gets also recognizable when looking at temperature‐dependent powder X‐ray diffraction patterns of Gd_2_[LiSi_3_N_5_O_2_] in Figure 4. The Rietveld refinement of the used powder sample indicated that the compound Gd_2_[LiSi_3_N_5_O_2_] is identified to represent the main phase of the reaction product with 90 wt% besides the minor phase (Gd_2_O_3_). The first signs of a thermal decomposition occur at 525 °C. Upon thermal decomposition between 525 and 700 °C, the original phase decomposes into SiO_2_ and LiGd_9_(SiO_4_)_6_O_2_ as the dominant products. These phases persist upon cooling and remain stable down to room temperature, indicating that the decomposition is irreversible within the investigated temperature range and that no reformation of the initial structure occurs under ambient conditions.

**FIGURE 4:**
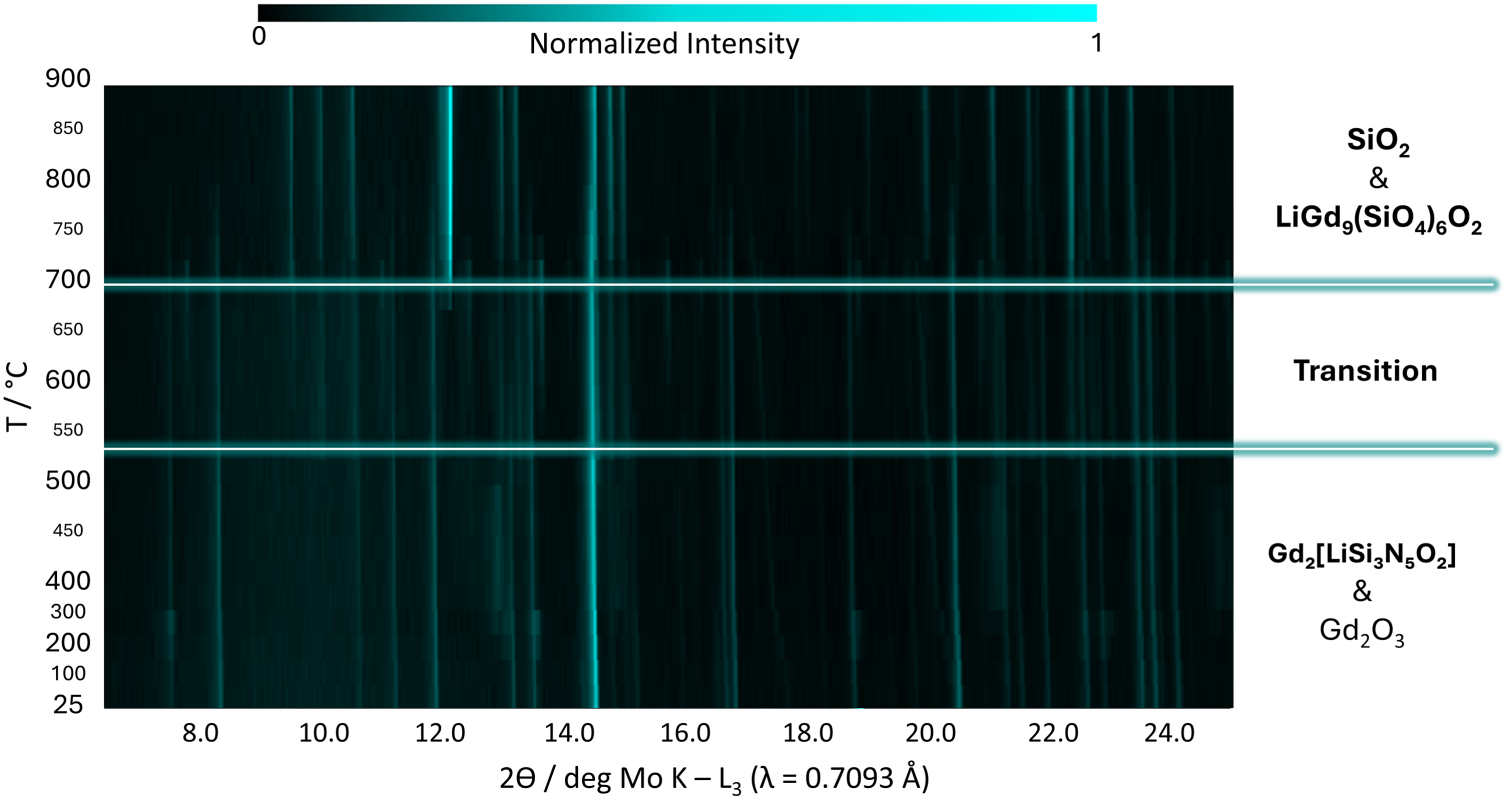
Temperature‐dependent powder X‐ray diffraction patterns of Gd_2_[LiSi_3_N_5_O_2_]. The temperature‐dependent systematic and continuous shift of the Bragg reflections indicates the cell expansion and/or structural distortion (<525 °C), while phase transitions are indicated by change of the Bragg reflections and their intensities (>525 °C).

To estimate the thermal behavior of Gd_2_[LiSi_3_N_5_O_2_], its unit cell parameters were determined in the temperature range from 25 to 525 °C, using Rietveld refinements of the powder diffraction patterns at the respective temperatures [52]. The relative changes of the cell parameters were calculated with respect to the reference temperature of 25 °C according to

(3) $$\frac{\Delta L}{L_{0}}=\frac{\left(L\left(T\right)-\;L_{0}\right)}{L_{0}}$$

where *L*(*T*) represents the cell parameter (*a* or *c*) at temperature *T* and *L*_0_ denotes the corresponding cell parameter at 25 °C. To obtain continuous temperature‐dependent trends and reduce the influence of experimental noise, the cell parameters were fitted using a linear (lin.) and quadratic (quad.) function of the form

(4) $$L_{\mathrm{lin}\text{.}}(T)=A+B\cdot T\;\mathrm{and}\;L_{\text{quad}.}(T)=A+B\cdot T+C\cdot T^{2}$$

where *A*, *B*, and *C* are fitting coefficients [53, 54]. The linear coefficients of thermal expansion (CTEs) were determined from the experimental data and the fitted functions. The temperature‐dependent thermal expansion coefficient was calculated according to

(5) $$\alpha \left(T\right)=\;\left(\frac{1}{L\left(T\right)}\right)\cdot \;\left(\frac{dL\left(T\right)}{dT}\right)$$

where *dL/dT* corresponds to the temperature derivative of the cell parameter [55, 56]. For the quadratic fit, the derivative was obtained by

(6) $$\frac{dL(T)}{dT}=\;B\;+\;2C\cdot T$$

The volumetric thermal expansion coefficient was derived analogously using the unit cell volume *V*(*T*):

(7) $$\alpha_{V\left(T\right)}=\;\left(\frac{1}{V\left(T\right)}\right)\cdot \;\left(\frac{dV\left(T\right)}{dT}\right)$$

The calculated values for the CTEs and quadratic and linear fits are shown in Table 7 with its coefficients of determination (*R* ^2^), and the corresponding data are shown graphically in Figure 5. Information about the fitting coefficients for the quadratic fit are listed in Table S15. For tetragonal symmetry, the relation ${\bar{\alpha }}_{V}=\;2{\bar{\alpha }}_{a}\;+\;{\bar{\alpha }}_{c}=2.1185\cdot 10^{-5}\;K^{-1}$ based on the quadratic fit was used as a consistency check. The agreement between directly calculated and derived values confirms the reliability of the analysis.

**TABLE 7:** Mean linear and volumetric coefficients of thermal expansion derived from linear fits (${\bar{\alpha }}_{\mathrm{lin}\text{.}}$) and quadratic fits (${\bar{\alpha }}_{\text{quad}.}$) for the cell parameters *a*, *c*, and the unit cell volume *V* of Gd_2_[LiSi_3_N_5_O_2_]:Eu^2+^. The coefficients of determination (*R* ^2^) are provided for both fits.

	${\bar{\alpha }}_{\mathrm{lin}\text{.}}$/**K** ^−1^	${\bar{\alpha }}_{\mathrm{quad}\text{.}}$/K^−1^	$R_{\mathrm{lin}\text{.}}^{2}$	$R_{\mathrm{quad}\text{.}}^{2}$
*a*	$6.7995\cdot 10^{-6}$	$6.7548\cdot 10^{-6}$	0.9964	0.9988
*c*	$7.7118\cdot 10^{-6}$	$7.6751\cdot 10^{-6}$	0.9991	0.9999
*V*	$2.1390\cdot 10^{-5}$	$2.1188\cdot 10^{-5}$	0.9978	0.9996

**FIGURE 5:**
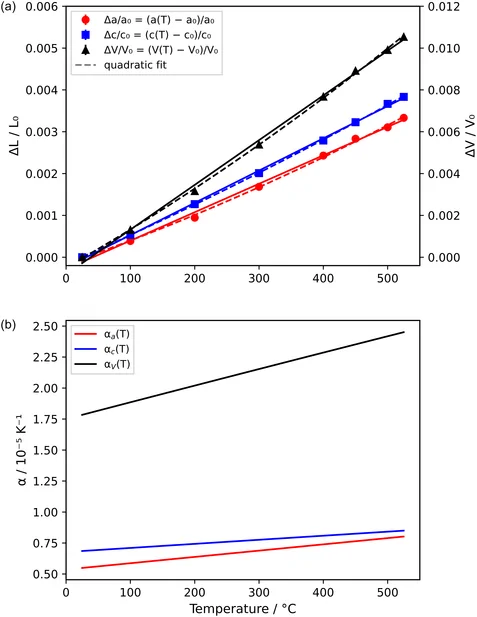
(a) Temperature dependence of the cell parameters *a* (red markers), *c* (blue markers), and *V* (black markers) expressed as relative thermal strain Δ*L*/*L*_0_ of Gd_2_[LiSi_3_N_5_O_2_]. The data are fitted using a linear (solid line) and quadratic fit (dashed line). (b) Temperature‐dependent linear thermal expansion coefficients *α*(*T*) obtained by differentiation of the quadratic functions.

The nearly linear temperature dependence of the cell parameters, as indicated by the minor improvement of the quadratic fit over the linear approximation, suggests that anharmonic contributions remain weak within the investigated temperature range. This behavior is consistent with a framework, in which thermal expansion is primarily governed by small‐amplitude rotations of rigid tetrahedral units in addition to increasing bond distances rather than structural rearrangement.

This assumption can be explained by the thermal behavior that shows that the connectivity governs the thermal expansion. Within the *a*–*b*‐plane, the layer is defined by strongly interconnected tetrahedral units forming *fünfer*‐rings, which impose significant geometric constraints and limit the flexibility. Consequently, the thermal expansion coefficient along this direction remains relatively low (${\bar{\alpha }}_{a}\;=6.75\;\cdot \;10^{-6}\;K^{-1}$). In contrast, the connectivity along the *c*‐axis involves mixed μ_2_‐N(Si,Li) linkages and a higher contribution of flexible [LiN_4_]‐tetrahedra. According to the inverse relationship between polyhedral thermal expansion and bond strength, originally established for oxides and applied here qualitatively, the weaker bonded [LiN_4_]‐units (Pauling bond strength *s* = 1/4) are considerably more compliant than the [SiN_4_]‐tetrahedra (*s* = 1) [57, 58]. In addition, their exceptionally large tetrahedral angle variance (*σ* ^2 ^≈ 310 deg^2^) shows that their geometry is imposed by the rigid silicate substructure, which makes them the preferred site for a thermally induced relaxation of bond angles and polyhedral tilting. As a result, a larger thermal expansion coefficient is observed along the *c*‐axis (${\bar{\alpha }}_{c}\;=7.68\;\cdot \;10^{-6}\;K^{-1}$). The moderate anisotropy (${\bar{\text{α}}}_{c}/{\bar{\text{α}}}_{a\;}\approx \;1.14$) reflects a balance between rigid and flexible structural units within a fully 3D network, rather than a layered structure. Therefore rigid [SiN_4_]‐tetrahedra act as backbone units, while the more distorted [SiN_3_O]‐ and especially [LiN_4_]‐tetrahedra accommodate structural strain and facilitate cooperative tilting mechanisms.

### 2.3 Structural Variability Across the Rare‐Earth Series

In analogy to the temperature‐induced cell response, the systematic structural change across the rare‐earth series can be interpreted as a chemically driven form of internal pressure, where gradual cation size reduction induces comparable cell adjustments within the same network. The systematic substitution of *RE* ^3+^ in *RE*_2_[LiSi_3_N_5_O_2_] (*RE* = Ce, Pr, Nd, Sm, Gd, Tb) and the change in the unit cell are shown in Figure 6, where the cell parameters *a* and *c*, unit cell volume *V*, polyhedral volume of the *RE* cation *V*_Poly_, and the average *RE*–*X* distances (<d_*RE*‐*X*_>) are shown versus the trivalent ionic radius of the rare‐earth elements. The exact values are listed in Table S16. Due to the combination of Rietveld refinements and single‐crystal X‐ray diffraction data, the corresponding structural parameters should be interpreted with caution. In particular, for the Pr‐containing compound, no phase‐pure powder sample could be obtained, and structural parameters are thus based exclusively on single‐crystal data (asterisked in Figure 6), which follow the expected trend consistently. A nearly linear correlation is observed between the ionic radius of *RE* ^3+^ and the cell parameters, demonstrating that the structure is highly adaptable and responds to the gradual size reduction of the incorporated rare‐earth ions. The average *RE*‐*X* bond lengths follow the same trend, decreasing from 2.5925(2) Å for Ce to 2.5362(2) Å for the Tb compound, which confirms a direct coupling between cation size and local coordination geometry.

**FIGURE 6:**
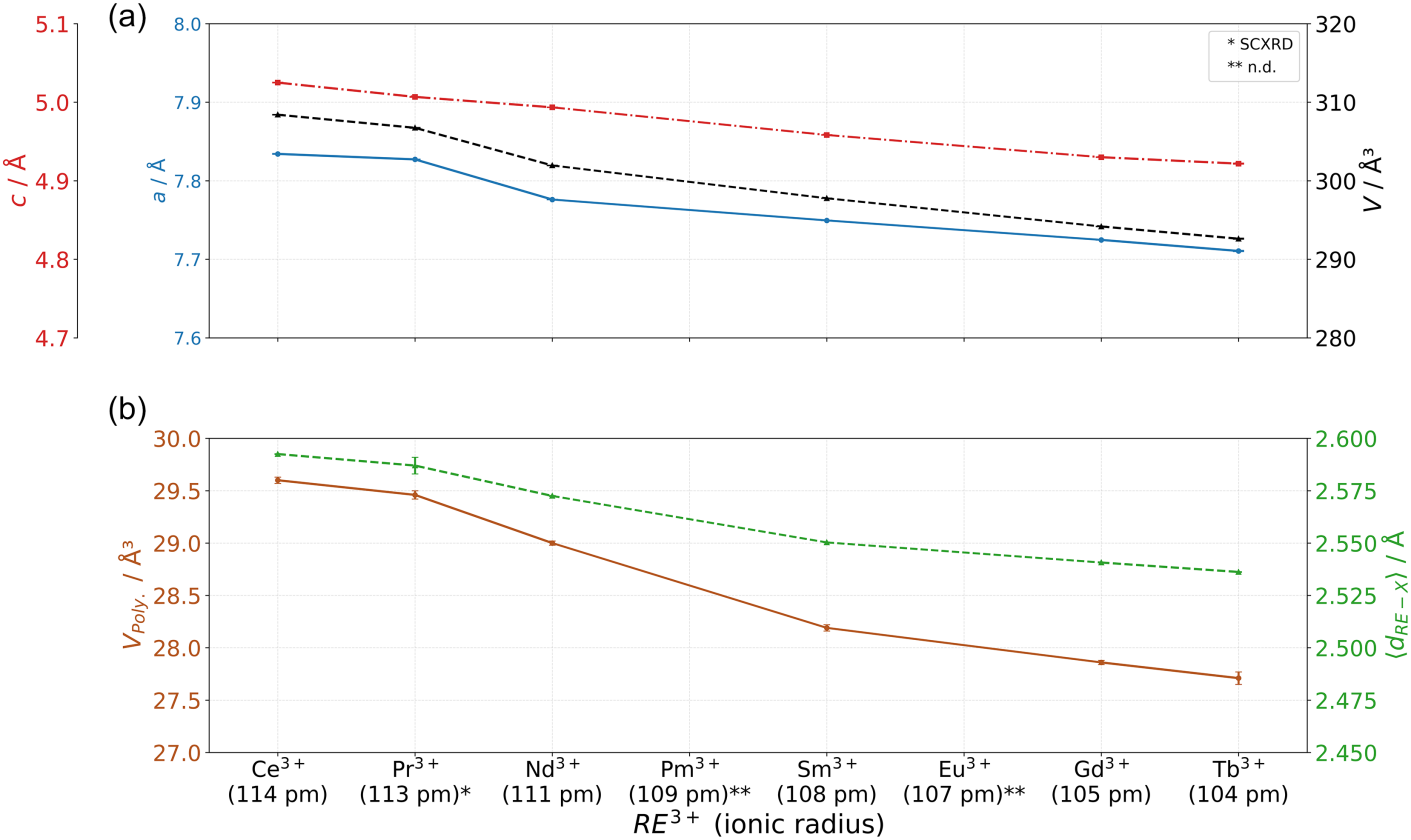
(a) Cell parameters *a*, *c*, and unit cell volume *V* versus the ionic radius of the rare‐earth elements. In (b), the polyhedral volume of the *RE* ^3+^ cation and the average *RE*–X distance versus ionic radius is plotted. The data are ordered from left to right by decreasing radii of the *RE* ^3+^ cations [59]. Error bars representing the standard deviations are included for each data point in the (a,b) but are not good visible due to their small magnitude. Parameters obtained from single‐crystal data are marked with an asterisk. Abbreviations: *RE* = rare‐earth element; *X* = ligand; *V* = cell volume; *V*_Poly. _= polyhedron volume; <d_*RE*–X_> = average distance between *RE* and X.

In addition to the rare‐earth coordination environment, the influence of the lanthanide contraction on the anionic substructure can be assessed by considering the average interatomic distances <$\bar{d}$_Si2‐N_>, <$\bar{d}$_Si1‐(N,O)_>, <$\bar{d}$_Li–N_>, and the interlayer spacing <d_A‐B_>. These values represent averaged bond lengths for the respective sites and are listed in Table S17. The Si‐centered tetrahedra exhibit only minor variations across the series. The [Si2N_4_]‐tetrahedra distances decrease slightly within experimental uncertainty (1.7307(1)–1.7485(1) Å), indicating that the [Si2N_4_]‐units behave as rigid structural building blocks. A deviation is observed for the Sm‐containing compound (1.7406(1) Å), which has a higher bond distance than the smaller and larger rare‍‐‍earth cations. Similarly, the mixed [Si1N_3_O]‐tetrahedra distances show only a slight decrease from Ce to Gd, followed by a somewhat higher value for Sm again, which may reflect increased distortion or experimental uncertainty rather than a systematic structural effect. Overall, these observations confirm that the silicate layer accommodates the lanthanide contraction primarily through tilting and distortion rather than bond compression.

The Li coordination environment shows a little higher sensitivity to the decreasing ionic radius. The [LiN_4_]‐tetrahedra distances decrease from approximately 2.043(3) Å (Ce, Pr) to 1.9965(2) Å (Tb), and they are consistent with a cooperative adjustment of the tetrahedral substructure. The interlayer distances <d_A‐B_> show an overall decreasing trend from 2.5125(2) Å (Ce) to approximately 2.4609(3) Å (Tb), reflecting a progressive contraction of the network along the stacking *c*‐direction.

The Si–N–Si angles show a systematic but weak dependence on the rare‐earth cations. An overview with the exact angels is shown in Table S18. For the mixed linkage (Si2–N^[2]^–Si1), values remain close to ~119°, with a slight decrease observed for smaller cations. The same observation accounts for the symmetric linkage (Si1–N^[2]^–Si1), which exhibits consistently larger angles around ~139°, with only minor variations across the series. The limited variation in both angle types further indicates that the distortion–tilt coupling mechanism is robust and only weakly disturbed by changes in the rare‐earth ionic radius. This suggests that the structure flexibility is primarily governed by the combination of slightly shorter bonds of the N‐bridged network and a weak change in tilting tetrahedra. A slight deviation from the general trend is observed for the Sm‐containing compound, where the Si1–N–Si1 angles are reduced compared to the other members of the series. This reduction correlates with the increased average bond distance of the Si‐tetrahedra, indicating enhanced tetrahedra tilting.

### 2.4 Luminescence

While cation size reduction and the associated substructure tilting, together with thermally induced structural responses, provide insight into the rigidity and flexibility of the network, they also establish the fundamental basis for its optical properties. In particular, the local coordination environment of the rare‐earth ions, together with the degree of structural distortion, is expected to influence the luminescence behavior. In previous work by our group, emission in Gd_2_[LiSi_3_N_5_O_2_]:Ce^3+^/Eu^2+^ highlighting the luminescent potential of this structural family was demonstrated [27]. In the present study, comparable 5d → 4f transitions are observed only for the Eu^2+^‐activated compound Tb_2_[LiSi_3_N_5_O_2_]:Eu^2+^, indicating that efficient activator incorporation and the resulting emission characteristics are dependent on its local structural environment. The crystallographic site occupied by Eu^2+^ cannot be exactly determined from the available data. Substitution at the *RE* ^3+^ site is considered most probable, because of local cell adaptation at low activator concentrations and charge compensation through anion substitution of oxygen instead of nitrogen in the immediate coordination environment, a mechanism formally analogous to related silicates [60, 61, 62]. Occupation of lithium positions represents also an alternative, as demonstrated, e.g., by Dutzler et al. for the compound RbKLi_2_[Li_3_SiO_4_]_4_:Eu^2+^ [28, 63, 64, 65, 66]. In analogy to melilite‐like structure types, in which the lithium site is not required for framework stability, local space for Eu^2+^ in the lithium plane through tetrahedral network relaxation or vacant lithium positions cannot be excluded.

In Figure 7, the photoluminescence emission (em) of a single grain of Tb_2_[LiSi_3_N_5_O_2_]:Eu^2+^ is shown. The emission was found to be homogeneously distributed throughout the entire powder sample. The emission maximum upon excitation with a 448 nm laser is located in the orange spectral region at *λ*_max_ = 618 nm (2.01 eV, 16,181 cm^−1^) with a full width at half‐maximum of the emission band of *fwhm* = 182 nm (0.57 eV, 4623 cm^−1^). The single grain was collected from a powder sample consisting of Tb_2_[LiSi_3_N_5_O_2_]:Eu^2+^ and Tb_2_O_3_ (34 wt%). The emission can be attributed to the phase Tb_2_[LiSi_3_N_5_O_2_]:Eu^2+^ in accordance with a sample showing that the emission maximum of a single grain of Tb_2_O_3_:Eu^2+^ is at 629 nm (1.97 eV, 15,898 cm^−1^) with a *fwhm* of 129 nm (0.40 eV). The emission spectrum upon excitation with blue light (*λ*_ex _= 448 nm) of Tb_2_O_3_:Eu^2+^ in addition to a Rietveld refinement of the phase‐pure powder is shown in Figures S4 and S5. The emission shown in Figure 7 differs from Tb_2_O_3_:Eu^2+^ in position (Δ*λ* = 11 nm) as well as in band width (0.57 vs. 0.40 eV) and is therefore assigned to Tb_2_[LiSi_3_N_5_O_2_]:Eu^2+^. As both bands overlap strongly and no independent phase characterization of the probed grain is available, a minor contribution of Tb_2_O_3_ inclusions to the spectrum cannot be excluded.

**FIGURE 7:**
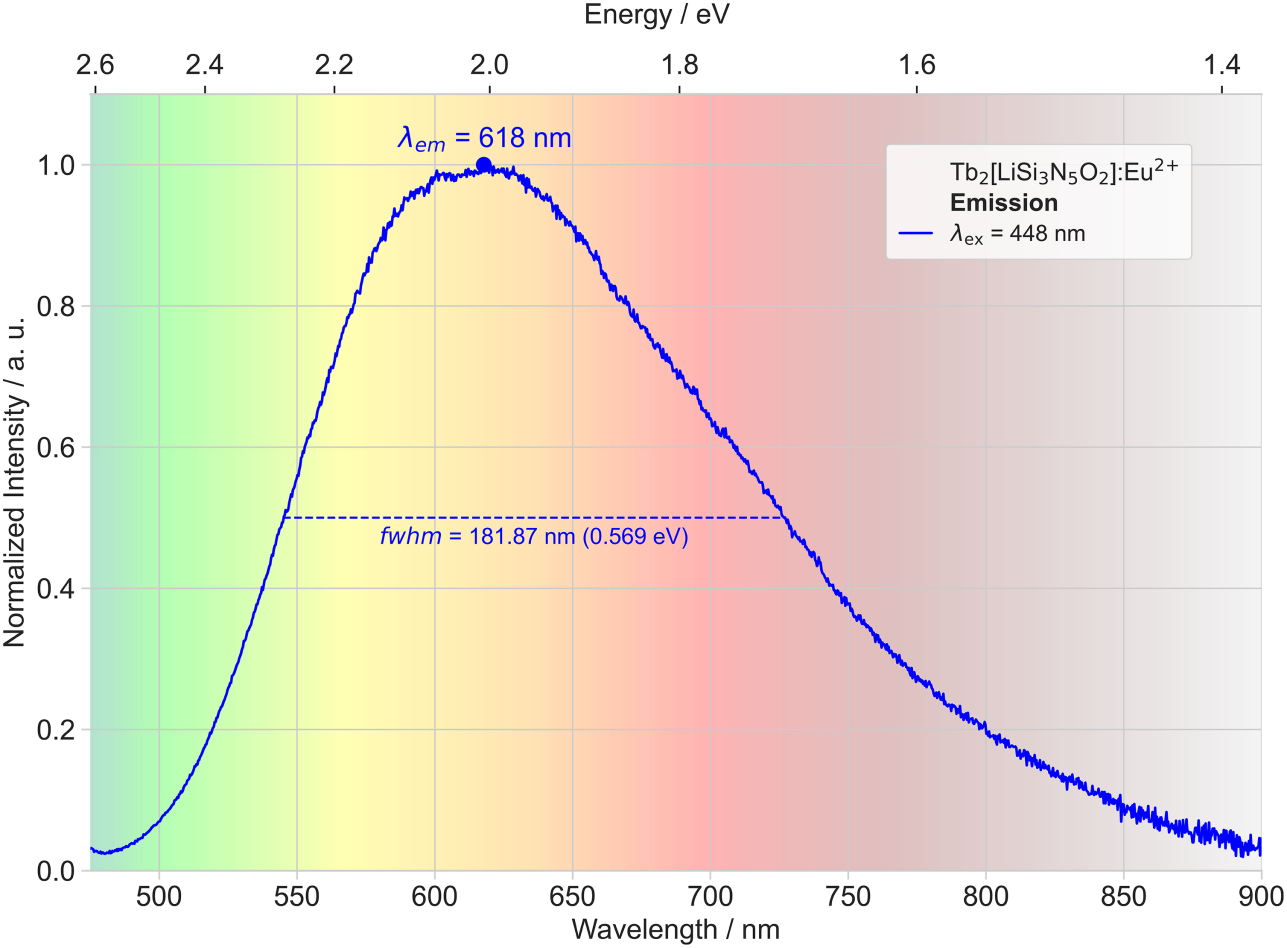
Emission spectrum of a single grain of Tb_2_[LiSi_3_N_5_O_2_]:Eu^2+^ upon excitation with blue light (*λ*_exc _= 448 nm). The emission spectrum was taken over 200 ms and averaged over 30 scans.

The crystallographic site of Eu^2+^ is not determined experimentally and the following considerations represent a hypothesis. The structure provides one *RE* site and a void within the *fünfer*‐rings, which is too small to host Eu^2+^ (*V* = 23.69 Å^3^ and ECoN = 1.81 in Gd_2_[LiSi_3_N_5_O_2_]:Eu^2+^), as the shortest distance to the neighboring N2 atom is 1.890(3) Å [27]. Eu^2+^ is therefore assumed to occupy the *RE* ^3+^ site. This aliovalent substitution requires local charge compensation, for instance, by O^2−^ on N^3−^ sites or by F^−^ on O^2−^ sites originating from the fluoride reactants. Different arrangements of Eu^2+^ and the compensating defect lead to a distribution of slightly different emitting centers, which is consistent with the large band width and the shoulder on the low‐energy side of the band.

The structural and optical data in Table 8 reflect a coherent response to the reduction in unit cell volume and coordination polyhedron volume from Gd_2_[LiSi_3_N_5_O_2_] to Tb_2_[LiSi_3_N_5_O_2_]. This is accompanied by a hypsochromic shift of the Eu^2+^ emission maximum from 644 to 618 nm, indicating that the emission behavior cannot be explained by the reduction of activator site volume and *RE–X* bond distances. A smaller activator site is expected to result in a stronger crystal field and thus in a bathochromic shift, which is not observed.

**TABLE 8:** Comparison of some key properties of the Eu^2+^ activated luminescence materials Gd_2_[LiSi_3_N_5_O_2_] and Tb_2_[LiSi_3_N_5_O_2_].

	Gd_2_[LiSi_3_N_5_O_2_]:Eu^2+^	Tb_2_[LiSi_3_N_5_O_2_]:Eu^2+^
Molecular mass/g mol^−1^	507.76	511.08
Cell parameter	@ RT	
*a*/Å	7.7247(2)	7.7106(6)
*c*/Å	4.9300(2)	4.9218(6)
*V*/Å^3^	294.18(2)	292.62(6)
*V*_Poly_/Å^3^	27.86(2)	27.71(6)
${\left({\mathrm{IR}}_{RE}+\;{\bar{\mathrm{IR}}}_{X}\right)}^{3}$/Å^3^	15.10	14.86
Δ*V*/Å^3^	12.76(2)	12.85(2)
ECoN (CN)	6.98(8)	6.76(8)
DI	0.049	0.057
*λ*_max_/nm	644	618
*fwhm/*eV	0.66	0.57

Abbreviations: Δ*V* = volume difference between $V_{\text{Poly}.}$ and ${\left({\mathrm{IR}}_{RE}+\;{\bar{\mathrm{IR}}}_{X}\right)}^{3}$, ${\left({\mathrm{IR}}_{RE}+\;{\bar{\mathrm{IR}}}_{X}\right)}^{3}$ = volume of ionic radii sum [59], *λ*_max_ = emission maxima, CN = coordination number, DI = distortion index, ECoN = effective coordination number, *fwhm =* full‐width at half‐maximum, *RE* = rare‐earth element, *V*_Poly._ = polyhedron volume, *V* = cell volume, *X* = ligand.

By analogy with the explanation proposed for Li_2_ *M*Si_2_N_4_ (*M* = Ca, Eu, Sr), the relevant quantity is not the absolute polyhedral volume but its difference to the ionic radii (IR) sum of the centering cation and ligands, expressed as

(8) $$\text{Δ}V=V_{\text{Poly}.}-{\left({\mathrm{IR}}_{RE}+\;{\bar{\mathrm{IR}}}_{X}\right)}^{3}$$

As Eu^2+^ substitutes for *RE* ^3+^, an increase in Δ*V* from Gd to Tb, as shown in Table 8, indicates that the activator site can be subjected to an effectively weaker local coordination environment, reducing ligand‐field splitting of the 5d levels and thus accounting for the observed hypsochromic shift [67].

A variation of the bond covalency may contribute as well to the observed direction. The smaller Tb^3+^ cation polarizes the shared anions more strongly, which would reduce the covalency of the activator bonds, increases the ionic potential (*z*/IR_*RE*_; *z* = cation charge, IR = ion radius) and thus reduces the centroid shift of the 5d levels (inductive effect) [68]. As Gd^3+^ and Tb^3+^ differ only slightly in ionic radius and electronegativity, this contribution is expected to be small. A reduced Stokes shift in the more confined environment of the Tb compound is consistent with both the hypsochromic shift and the smaller band width (0.57 vs. 0.66 eV).

In addition, the Gd_2_[LiSi_3_N_5_O_2_]:Eu^2+^ and Tb_2_[LiSi_3_N_5_O_2_]:Eu^2+^ bands are broad and probably comprise different centers, so that different relative contributions of these centers can shift the maxima as well. The parameter Δ*V* (Equation (8)) is therefore regarded as one possible descriptor and not as the only origin of the shift. A conclusive interpretation requires phase‐pure samples as well as low‐temperature and time‐resolved luminescence measurements of both samples.

Beyond the intrinsic emission, the luminescence properties of differently activated *RE*_2_[LiSi_3_N_5_O_2_] compounds were systematically investigated. All compositions were subjected to activation attempts using Eu^2+^ and Ce^3+^. The successful incorporation was only achieved for the compounds Gd_2_[LiSi_3_N_5_O_2_]:Eu^2+^/Ce^3+^ and Tb_2_[LiSi_3_N_5_O_2_]:Eu^2+^. Nevertheless, luminescence is observed in samples synthesized without intentional activation, indicating that intrinsic 4f → 4f transitions of the lanthanide ions contribute to the emission [69]. Upon Ce^3+^ activation, the emission intensity of these 4f → 4f transitions is generally enhanced, suggesting an efficient energy transfer from Ce^3+^ to the host rare‐earth ions. An exception is observed for Gd_2_[LiSi_3_N_5_O_2_]:Ce^3+^, where a blue emission of the Ce^3+^ 5d → 4f transitions is reported [27]. This behavior is consistent with the high energetic position of the excited 4f states of Gd^3+^, which prevents efficient energy transfer from Ce^3+^ to the host ion [70, 71].

In order to provide further insight into the intrinsic emission behavior, single‐crystals and selected grains were isolated from powder samples of Tb_2_[LiSi_3_N_5_O_2_] and Nd_2_[LiSi_3_N_5_O_2_], both of which exhibit the expected 4f → 4f transitions of the respective rare‐earth ions [69]. No additional emission bands attributable to side phases were detected under the applied experimental conditions, thereby confirming that the observed luminescence originates from the title compounds. The luminescences of the investigated samples are shown in Figure 8.

**FIGURE 8:**
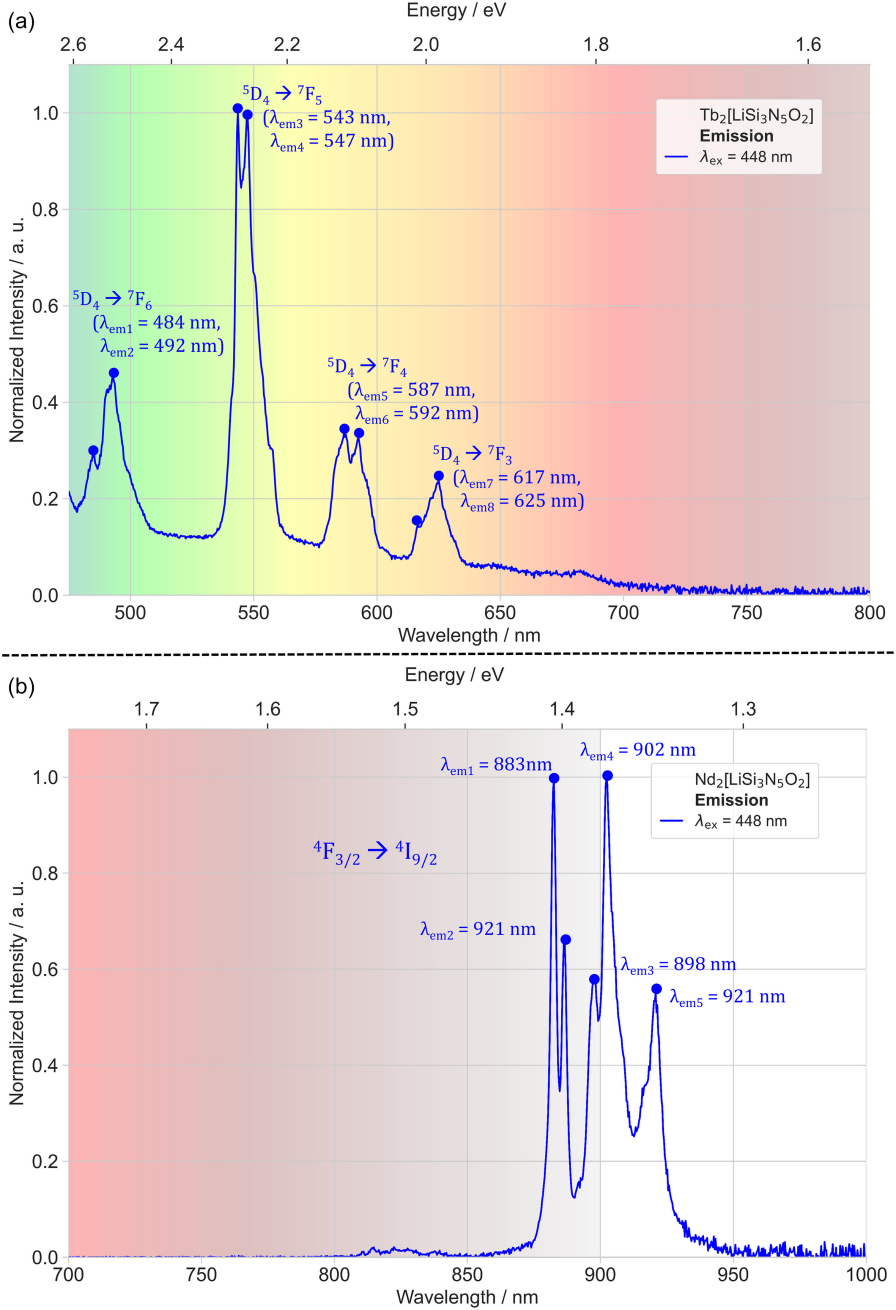
Emission spectra upon excitation with blue light (*λ*_exc _= 448 nm) of single grains of Tb_2_[LiSi_3_N_5_O_2_] (top) and Nd_2_[LiSi_3_N_5_O_2_] (bottom). The emission spectra were taken over 1 s and averaged over five scans.

For all compounds in the system *RE*_2_[LiSi_3_N_5_O_2_] (*RE* = Ce, Pr, Nd, Sm, Gd, Tb), the *RE* ^3+^ ions occupy the Wyckoff‐position 4*e* with site symmetry *m* (*C*_s_). The rare‐earth site is embedded in a strongly distorted antiprismatic coordination environment formed by O^2−^ and N^3−^ ligands. This mixed‐anionic coordination gives rise to an anisotropic crystal field, in which a subset of shorter *RE*–N and *RE*–O bonds dominate the local electrostatic potential. The crystal field acting on the 4f manifold is weak compared to spin–orbit coupling. Nevertheless, it lifts the degeneracy of the J‐multiplet states via Stark splitting into a set of crystal‐field sublevels determined by the local symmetry. The low site symmetry can further introduce odd‐parity perturbations of the crystal‐field potential, which may partially relax the parity selection rule and enhance the intensity of electric‐dipole forbidden 4f–4f transitions [70, 72, 73, 74, 75, 76].

The emission spectrum of Tb^3+^ (4f^8^) is dominated by the characteristic ^5^D_4_ →^7^F_J_ transitions. Each transition appears as a pair of closely spaced lines, resulting in a total of eight resolved emission peaks [70, 72]. Although the low site symmetry (*C*_s_) formally allows a complete lifting of the (2*J *+ 1)‐fold degeneracy into individual Stark levels, only a limited number of transitions is experimentally resolved. This is attributed to strongly varying oscillator strengths, such that only a subset of Stark components exhibits sufficient intensity. In addition, inhomogeneous broadening arising from a distribution of local crystal‐field environments, together with electron–phonon coupling leads to partial overlap and merging of closely spaced Stark transitions. The observed doublet‐like features therefore reflect moderate crystal‐field splitting in a highly anisotropic coordination environment [70, 72, 73].

The luminescence behavior of Nd^3+^ (4f^3^) is governed by Kramers degeneracy, which ensures that each crystal field level remains at least twofold degenerate in the absence of a magnetic field. For Nd^3+^, the ^4^I_9/2_ ground multiplet splits into five Kramers doublets and the ^4^F_3/2_ excited state into two Kramers doublets, yielding a maximum of 10 electric dipole transitions, all of which are group‐theoretically allowed in the present *C*_s_ site symmetry. Upon excitation at 448 nm, the emission originates from the ^4^F_3/2 _→ ^4^I_9/2_ transition and emits multiple bands in the NIR region. Experimentally, five partially overlapping maxima are resolved at room temperature, consistent with the expected splitting pattern when thermal population of multiple ground‐state Stark levels, differential transition intensities governed by crystal field and inhomogeneous line broadening are taken into account [70, 73, 76, 77].

## 3 Conclusions

This study reports the synthesis and comprehensive characterization of the isotypic series *RE*_2_[LiSi_3_N_5_O_2_] (*RE* = Ce, Pr, Nd, Sm, Tb), establishing this compound class as a structurally coherent and versatile luminescent family of rare‐earth oxonitridolithosilicates. The single‐crystal X‐ray diffraction and Rietveld refinement confirms that all members adopt the tetragonal crystal system and are isotypic to the previously reported crystal structure of Gd_2_[LiSi_3_N_5_O_2_]. The cell parameters decrease across the series is fully consistent with the lanthanoid contraction. The absence of structural phase transitions across the series at room temperature confirms that the structure adapts the decrease in ionic radius through a strain response through rigid and flexible units. The [SiN_4_]‐tetrahedra form a rigid backbone through N^[2]^ linkages, while the [SiN_3_O]‐tetrahedra act as local distortion buffers due to the presence of terminal oxygen ligands. The [LiN_4_]‐tetrahedra introduce additional flexibility via mixed μ_2_‐N(Si,Li) connections and the high distortion.

The optical characterization reveals that the luminescence properties are strongly host‐dependent and reflect the interplay between local site symmetry, coordination geometry, and electronic configuration of the *RE* ^3+^ ion. For the Eu^2+^‐activated compound Tb_2_[LiSi_3_N_5_O_2_], a broadband 5d → 4f emission is observed at *λ*_max_ = 618 nm (2.01 eV, 16,181 cm^−1^) with *fwhm* = 0.57 eV (4623 cm^−1^). A direct comparison with Gd_2_[LiSi_3_N_5_O_2_]:Eu^2+^ (*λ*_max_ = 644 nm, *fwhm* = 0.66 eV) reveals a hypsochromic shift and simultaneous narrowing of the emission band. For the stoichiometric *RE* ^3+^ constituents Tb^3+^ and Nd^3+^, characteristic intra‐configurational f–f emissions are observed. Tb^3+^ exhibits the characteristic ^5^D_4 _→ ^7^F_J_ manifold, with the large number of Stark‐split components only partially resolved due to inhomogeneous broadening in the strongly distorted coordination environment. Nd^3+^ displays NIR emission from the ^4^F_3/2 _→  ^4^I_9/2_ state, consistent with Kramers degeneracy [73, 76].

Besides these central findings some limitations of the present system have to be considered. The powder samples are not phase‐pure, and the luminescence data were therefore recorded on single grains. The site of Eu^2+^ and the origin of the hypsochromic shift from Gd to Tb are not determined directly but are derived by exclusion. The interatomic distances rule out the lithium site and the void within the *fünfer‐*rings and leave the *RE* ^3+^ site as the most suitable position. In the same way, compression of the activator site is ruled out as the origin of the hypsochromic shift, as it would act in the opposite direction. Nevertheless, the occupation of the *RE* ^3+^ site and the parameter Δ*V* provide a consistent description of the structural and spectroscopic data. The applicability of Δ*V*, originally proposed for Li_2_ *M*Si_2_N_4_ (*M* = Ca, Eu, Sr), to a second structure family makes this parameter a descriptor of interest for emission shifts that cannot be explained by the polyhedral volume alone [67].

In summary, *RE*_2_[LiSi_3_N_5_O_2_] (*RE* = Ce, Pr, Nd, Sm, Gd, Tb) represents a compound group for the systematic study of rare‐earth luminescence in a mixed anionic coordination environment and as a model system for understanding of structure–property relations in (rare‐earth) oxonitridolithosilicates. The present series shows that structural trends across the lanthanide contraction are well‐defined and expanding the series through variation of the alkali metal, O/N ratio, or *RE*/Si stoichiometry may yield further insights. Beyond luminescence, the structural versatility and chemical tunability of (oxonitrido)silicates make this compound class relevant for a broad range of technological applications, including, e.g., ionic conductors or thermally stable host materials [24, 26, 42].

## 4 Experimental Section

### 4.1 Synthesis of *RE*_2_[LiSi_3_N_5_O_2_] (*RE* = Ce, Pr, Nd, Sm, Gd, Tb) (With Eu^2+^‐Activation)

High‐temperature solid‐state reactions in arc‐welded tantalum tubes under an argon atmosphere were employed to obtain *RE*_2_[LiSi_3_N_5_O_2_] (*RE* = Ce, Pr, Nd, Sm, Gd, Tb). Therefore, the reactants Si_3_N_4_ (UBE SN‐E10, >99.9%), SiO_2_ (Alfa Aesar, >99.9%), Li_3_N (Alfa Aesar, 99.4%) and *RE*F_3_ (*RE* = Ce, Pr, Nd, Sm, Gd, Tb; Strem Chemicals, 99.9%) were weighed according to the stoichiometric ratio 2:3:7:6 and mixed inside a glovebox (argon atmosphere, UniLab Plus Glove Box Workstation, MBraun, Garching, Germany). The reaction of the *RE*F_3_ with Li_3_N is a metathesis reaction, in which the formation of LiF provides the thermodynamic driving force. The activation of Tb_2_[LiSi_3_N_5_O_2_] with Eu^2+^ was achieved using 1–2 wt% EuF_2_ among the reactants (Alfa Aesar, 99.9%). Under inert gas atmosphere (Ar 5.0, Messer Austria GmbH), the compounds were ground in an agate mortar using a pestle and filled in tantalum ampoules. The tantalum ampoules were sealed via argon arc welding and placed inside of a silica glass ampoule containing a low‐pressure inert gas atmosphere. The temperature profile of the synthesis in a tube furnace consisted of a heating ramp with a rate of 240 °C/h up to 940 °C, a dwelling time of 12 h, and subsequently a cooling ramp with a rate of 6 °C/h to 500 °C and 18 °C/h to 150 °C. Afterward, the tube furnace was switched off. Once the ampoule had cooled to room temperature, the sample could be handled under air without any measurable change over a period of several months. The use of EuF_2_ as a reactant resulted in powders exhibiting an orange luminescence upon UV excitation (Gd_2_[LiSi_3_N_5_O_2_]:Eu^2+^ and Tb_2_[LiSi_3_N_5_O_2_]:Eu^2+^).

### 4.2 Microscopy

The examination of samples and the preparation of single crystals was conducted using a Leica M125 stereomicroscope with the external light sources SCHOTT KL1500 LCD and KL2500 LCD, in conjunction with a Leica DFC420 camera.

### 4.3 Single‐Crystal X‐Ray Diffraction

In the context of structural investigations based on single crystals, a Bruker D8 QUEST diffractometer (Mo K‐L_2,3_ radiation, *λ* = 0.71073 Å; Cu K‐L_2,3_ radiation_,_ *λ *= 1.54178 Å) was employed, equipped with a PHOTON III detector. The multiscan absorption correction and subsequent data processing were conducted utilizing the SAINT and SADABS software tools, both implemented in APEX [34, 78, 79]. The crystal structures were solved with SHELXT using intrinsic phasing and refined with SHELXL, both algorithms implemented in the OLEX2 suite [80, 81, 82]. The crystal structures and the determined symmetries were checked in Platon using the ADDSYM routine [83, 84]. The crystal structures were visualized using DIAMOND 4.6.4 [85]. Further crystallographic data can be accessed via the Cambridge Crystallographic Data Centre (CCDC) and the Fachinformationszentrum Karlsruhe Access Structures Service at www.ccdc.cam.ac.uk/structures under the deposition numbers 2563255 (Pr_2_[LiSi_3_N_5_O_2_]), 2563142 (Nd_2_[LiSi_3_N_5_O_2_]), 2496822 (Gd_2_[LiSi_3_N_5_O_2_]) and 2563143 (Tb_2_[LiSi_3_N_5_O_2_]).

### 4.4 Temperature‐Dependent Powder X‐Ray Diffraction

For the collection of temperature‐dependent powder X‐ray diffraction data, the abovementioned STOE Stadi P powder diffractometer was equipped with a STOE resistance graphite furnace. The sample was filled into a quartz capillary with an outer diameter of 0.3 mm (Hilgenberg, Malsfeld) and heated from 25 to 1000 °C in steps of 100 °C till 400 °C and then in steps of 25–1000 °C. The cooling of the sample followed the same ramps. Between two steps, a heating and cooling rate of 50 °C/min was used. One last measurement was collected at room temperature after switching off the furnace.

### 4.5 Luminescence Spectroscopy

The emission spectra of single‐crystals and grains, mounted on a glass slide, were measured using a setup comprising a blue laser diode with *λ *= 448 nm (THORLABS, Newton, NJ, US) and a CCD Detector (AVA AvaSpec 2048, AVANTES, Apeldoorn, Netherlands) in conjunction with the software AVA AvaSoft (version 7). The wavelength recorded covered a range of 420–900 nm, with a step size of 0.330 nm.

## Funding

Support by the Federal Ministry for Economic Affairs and Climate Action on the basis of a decision by the German Bundestag and by the Bavarian Ministry of State for Economic Affairs, Regional Development and Energy as well as by the European Union – NextGenerationEU for the Important Project of Common European Interest on microelectronics and communication technologies (IPCEI ME/CT) ‐ OptoSuRe (16IPCEI221) is gratefully acknowledged.

## Conflicts of Interest

The authors declare no conflicts of interest.

## Supporting information

Detailed crystallographic data, structure refinements, Wyckoff‐positions, atomic coordinates, equivalent displacement parameters, and anisotropic displacement parameters for *RE*_2_[LiSi_3_N_5_O_2_] (*RE* = Pr, Gd, Tb) (Table S1 to S7). Results and Figures of the Rietveld refinement of the powder samples for *RE*_2_[LiSi_3_N_5_O_2_] (*RE* = Ce, Sm, Gd, Tb) (Table S8–S10 and Figure S1–S3). Calculated bond valences using BLBS (∑V) and CHARDI‐2015 (∑Q) for *RE*_2_[LiSi_3_N_5_O_2_] (*RE *= Pr, Gd, Tb) (Table S11–S12). Partial MAPLE values and MAPLE sums for *RE*_2_[LiSi_3_N_5_O_2_] (*RE *= Pr, Nd, Gd, Tb) (Table S13). Selected interatomic distances for Nd_2_[LiSi_3_N_5_O_2_] (Table S14). Fitting coefficients A, B, C for the quadratic fit for the temperature‐dependent lattice parameters in Gd_2_[LiSi_3_N_5_O_2_] (Table S15). Cell parameters, unit cell volume, polyhedral volume of the *RE* cation, average *RE*–*X* distance, interatomic distances, layer spacings and selected interatomic angles of the compounds *RE*_2_[LiSi_3_N_5_O_2_] (*RE* = Ce, Pr, Nd, Sm, Gd, Tb) (Table S17 and S18). Selected optical data of the emission spectrum measurement of Tb_2_O_3_:Eu^2+^ and the Rietveld refinement of the measured powder sample (Figure S4 and S5).

## Data Availability

Further crystallographic data can be accessed via the Cambridge Crystallographic Data Centre (CCDC) and the Fachinformationszentrum Karlsruhe Access Structures Service at www.ccdc.cam.ac.uk/structures under the deposition numbers 2563255 (Pr_2_[LiSi_3_N_5_O_2_]), 2563142 (Nd_2_[LiSi_3_N_5_O_2_]), 2496822 (Gd_2_[LiSi_3_N_5_O_2_]) and 2563143 (Tb_2_[LiSi_3_N_5_O_2_]).
