# Supplementary material for: Crystal Structures and Luminescence Properties of the Oxonitridolithosilicates RE 2[LiSi3N5O2] (RE = Ce, Pr, Nd, Sm, Gd, Tb)
Source: ChemistryOpen. 2026 Nov 8;15(11):e70329. doi: 10.1002/open.70329 (PMC13745805; doi:10.1002/open.70329)
Supplement: Supplementary file 1 [file OPEN-15-e70329-s001.pdf]

Supporting Information

to

Crystal Structures and Luminescence Properties of the Oxonitridolithosilicates $RE_2[LiSi_3N_5O_2]$ (RE = Ce, Pr, Nd, Sm, Gd, Tb)

Table S1: Detailed crystallographic data and structure refinements of $RE_2[LiSi_3N_5O_2]$ (RE = Pr, Nd, Tb) and $Gd_2[LiSi_3N_5O_2]:Ce^{3+}$.[1]

**Parameter**	**Value**			
Empirical formula	$RE_2[LiSi_3N_5O_2]$			
RE	Pr	Nd	Gd[1]	Tb
Molecular mass / g mol^{-1}	475.08	481.74	507.76	511.10
Crystal system	tetragonal			
Space group	$P\bar{4}2_1m$ (no. 113)			
Single-crystal diffractometer	Bruker D8 QUEST PHOTON III C14			
Radiation	Mo K-L$_{2,3}$ (λ = 0.71073 Å)			Cu K-L$_{2,3}$ (λ = 1.54178 Å)
a / Å	7.8272(1)	7.7998(1)	7.7178(1)	7.6915(1)
c / Å	5.0068(1)	4.9909(2)	4.9324(1)	4.9301(1)
V /Å^3	306.74(2)	303.63(2)	293.80(2)	291.66(2)
Z	2			
Calculated density / g cm^{-3}	5.144	5.269	5.740	5.820
Crystal size / mm	0.05 · 0.05 · 0.05	0.15 · 0.15 · 0.15	0.049 · 0.028 · 0.021	0.01 · 0.02 · 0.03
Temperature / K	293	293	301	296
Absorption coefficient / mm^{-1}	16.232	17.452	22.938	123.286
F (000) / e	428	432	448	452
2θ-range / deg	3.68 – 42.22	3.69 – 36.40	3.73 – 39.99	5.75 – 47.98
Range in *hkl*	$-14<h<14$; $-14<k<14$; $-9<l<9$	$-9<h<13$; $-12<k<12$; $-8<l<8$	$-14<h<14$; $-14<k<14$; $-9<l<9$	$-7<h<3$; $-7<k<4$; $-2<l<4$
Reflections total / independent	17498 / 1166	7341 / 797	9254 / 1045	877 / 148
R_{int}	0.0306	0.0274	0.0580	0.0973
Reflections [$I \geq 2\sigma(I)$]	1118	780	991	121
R_σ	0.0123	0.0157	0.0157	0.0740
Data / ref. parameters	1166 / 38	797 / 38	1045 / 36	148 / 28
Absorption correction	multi-scan (SADABS-2016/2)[2]			
Goodness of fit on F^2	1.130	1.125	1.019	1.233
Flack parameter	0.22(3)	0.25(2)	0.00(2)	0.1710(2)
Final R_1 / wR_2 [$I \geq 2\sigma(I)$]	0.0154 / 0.0363	0.0117 / 0.0268	0.0184 / 0.0425	0.0727 / 0.1340
Final R_1 / wR_2 (all data)	0.0171 / 0.0373	0.0121 / 0.0270	0.0200 / 0.0432	0.0956 / 0.1456
Largest diff. peak/ hole / e·Å^{-3}	1.384 / -1.420	0.750 / -0.841	2.201 / -1.106	0.518 / -0.458

Table S2: Wyckoff-positions, atomic coordinates and equivalent displacement parameters for $Pr_2[LiSi_3N_5O_2]$ (standard deviations in parentheses).

Atom	Wyckoff-position	*x*	*y*	*z*	U_{eq} / Å^2
Pr1	4*e*	0.16079(2)	*x*+½	0.50217(5)	0.00883(5)
Si1	4*e*	0.6435(2)	*x*+½	0.9401(2)	0.0068 (2)
Si2	2*a*	0	0	0	0.0061(2)
O1	4*e*	0.6423(3)	*x*+½	0.2737(6)	0.0101(4)
N1	8*f*	0.0803(3)	0.1609(3)	0.2049(4)	0.0089(3)
N2	2*c*	0	½	0.1759(8)	0.0065(6)
Li1	2*b*	0	0	½	0.024(2)

Table S3: Anisotropic displacement parameters U_{ij} (Å^2) of $Pr_2[LiSi_3N_5O_2]$ (standard deviations in parentheses).

Atom	U_{11}	U_{22}	U_{33}	U_{12}	U_{13}	U_{23}
Pr1	0.00967(6)	0.00967(6)	0.00715(6)	-0.00296(4)	-0.00095(6)	-0.00095(6)
Si1	0.0069(2)	0.0069(2)	0.0066(3)	-0.0007(3)	0.00004(19)	0.00004(19)
Si2	0.0059(2)	0.0059(2)	0.0065(4)	0.00000	0.00000	0.00000
O1	0.0094(6)	0.0094(6)	0.0115(10)	0.0004(9)	-0.0016(6)	-0.0016(6)
N1	0.0092(8)	0.0082(8)	0.0093(7)	0.0006(6)	-0.0018(6)	-0.0017(7)
N2	0.0065(9)	0.0065(9)	0.0066(13)	0.0013(11)	0.00000	0.00000
Li1	0.023(3)	0.023(3)	0.025(5)	0.00000	0.00000	0.00000

Table S4: Anisotropic displacement parameters U_{ij} (Å^2) of $Nd_2[LiSi_3N_5O_2]$ (standard deviations in parentheses).

Atom	U_{11}	U_{22}	U_{33}	U_{12}	U_{13}	U_{23}
Nd1	0.00834(6)	0.00834(6)	0.00743(7)	-0.00221(4)	-0.00112(6)	-0.00112(6)
Si1	0.0061(2)	0.0061(2)	0.0061(3)	-0.0001(3)	0.00012(18)	0.00012(18)
Si2	0.0048(2)	0.0048(2)	0.0051(4)	0.00000	0.00000	0.00000
O1	0.0094(7)	0.0094(7)	0.0097(9)	-0.0003(9)	-0.0013(6)	-0.0013(6)
N1	0.0081(8)	0.0061(8)	0.0080(7)	-0.0002(7)	-0.0016(6)	-0.0013(7)
N2	0.0064(10)	0.0064(10)	0.0061(14)	0.0019(12)	0.00000	0.00000
Li1	0.022(3)	0.022(3)	0.018(4)	0.00000	0.00000	0.00000

Table S5: Wyckoff-positions, atomic coordinates, and equivalent displacement parameters for $Gd_2[LiSi_3N_5O_2]:Ce^{3+}$ (standard deviations in parentheses).[1]

Atom	Wyckoff-position	*x*	*y*	*z*	U_{eq} / Å^2
Gd1	4*e*	0.16055(2)	*x*+½	0.50300(6)	0.01037(5)
Si1	4*e*	0.6441(2)	*x*+½	0.9379(3)	0.0075(2)
Si2	2*a*	0	0	0	0.0066(2)
O1	4*e*	0.6423(3)	*x*+½	0.2759(7)	0.0097(5)
N1	8*f*	0.0823(4)	0.1606(4)	0.2091(6)	0.0091(4)
N2	2*c*	0	½	0.183(2)	0.0083(8)
Li1	2*b*	0	0	½	0.024(3)

Table S6: Wyckoff-positions, atomic coordinates, and equivalent displacement parameters for $Tb_2[LiSi_3N_5O_2]$ (standard deviations in parentheses).

Atom	Wyckoff-position	*x*	*y*	*z*	U_{eq} / Å^2
Tb1	4*e*	0.1620(4)	*x*+½	0.503(2)	0.04(2)
Si1	4*e*	0.646(2)	*x*+½	0.941(4)	0.04(2)
Si2	2*a*	0	0	0	0.04(2)
O1	4*e*	0.639(5)	*x*+½	0.27(2)	0.04(2)
N1	8*f*	0.084(5)	0.170(6)	0.201(9)	0.02(2)
N2	2*c*	0	½	0.18 (2)	0.01(2)
Li1	2*b*	0	0	½	0.00(3)

Table S7: Anisotropic displacement parameters U_{ij} (Å^2) of $Tb_2[LiSi_3N_5O_2]$ (standard deviations in parentheses).

Atom	U_{11}	U_{22}	U_{33}	U_{12}	U_{13}	U_{23}
Tb1	0.035(16)	0.035(16)	0.031(16)	-0.002(3)	-0.001(4)	-0.001(4)
Si1	0.038(19)	0.038(19)	0.03(2)	-0.012(13)	-0.002(10)	-0.002(10)
Si2	0.031(19)	0.031(19)	0.05(2)	0.00000	0.00000	0.00000
O1	0.05(3)	0.05(3)	0.02(4)	-0.03(4)	0.00(2)	0.00(2)

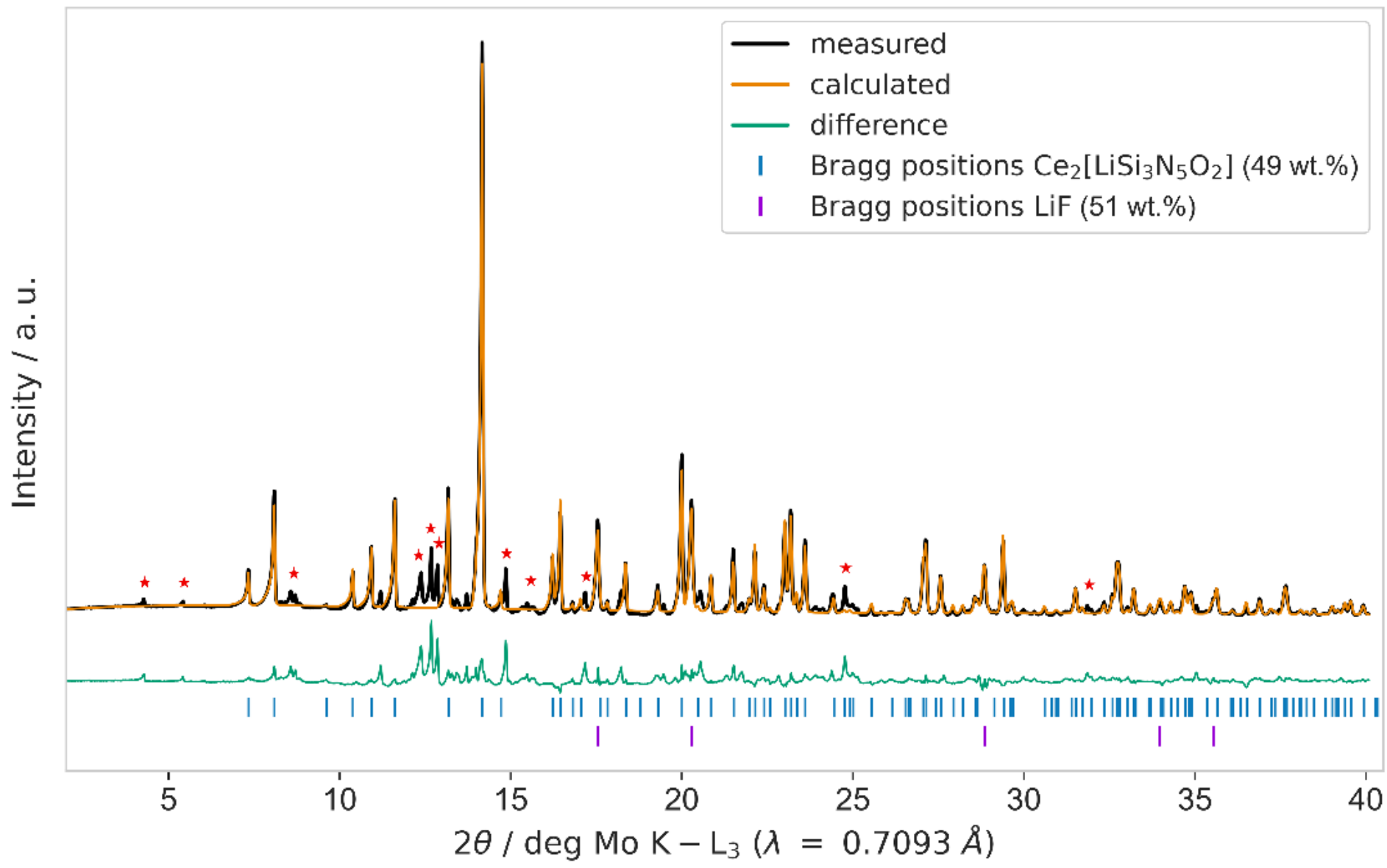

Figure S1: Rietveld fit of the measured powder diffraction data (black line) of $Ce_2[LiSi_3N_5O_2]$, the calculated pattern based on the theoretical reflections of the phases (orange line), and the difference plot (green line). Tick marks represent the theoretical reflection position of $Ce_2[LiSi_3N_5O_2]$ (blue tick marks; 49 wt.%) and LiF (purple tick marks; 51 wt.%). Reflections that are not identified are asterisked (red).

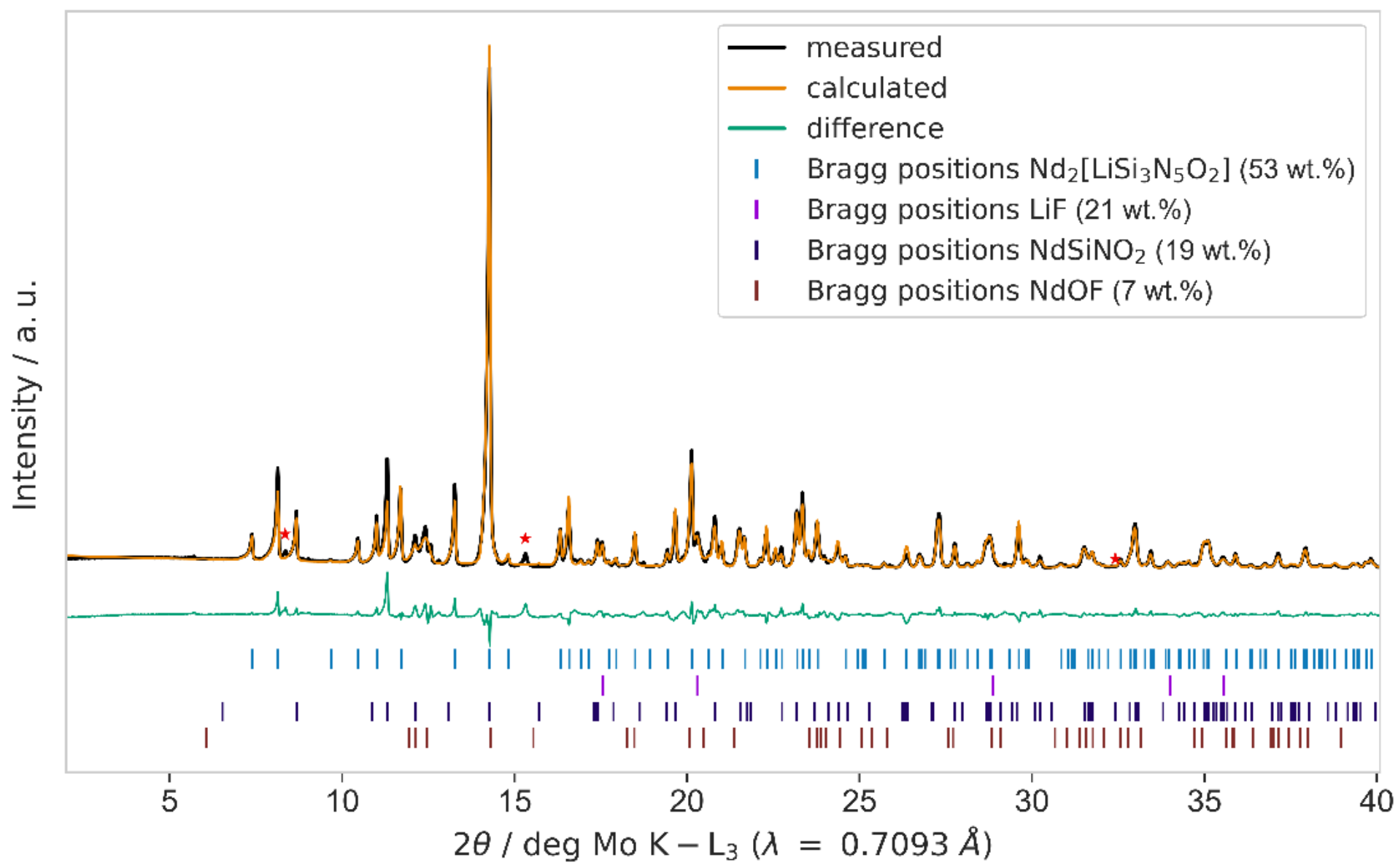

Figure S2: Rietveld fit of the measured powder diffraction data (black line) of $Nd_2[LiSi_3N_5O_2]$, the calculated pattern based on the theoretical reflections of the phases (orange line), and the difference plot (green line). Tick marks represent the theoretical reflection position of $Nd_2[LiSi_3N_5O_2]$ (blue tick marks; 53 wt.%) and of the side phases LiF (purple tick marks; 21 wt.%), $NdSiNO_2$ (dark blue tick marks; 19 wt.%) and NdOF (brown tick marks; 7 wt.%). Reflections that are not identified are asterisked (red).

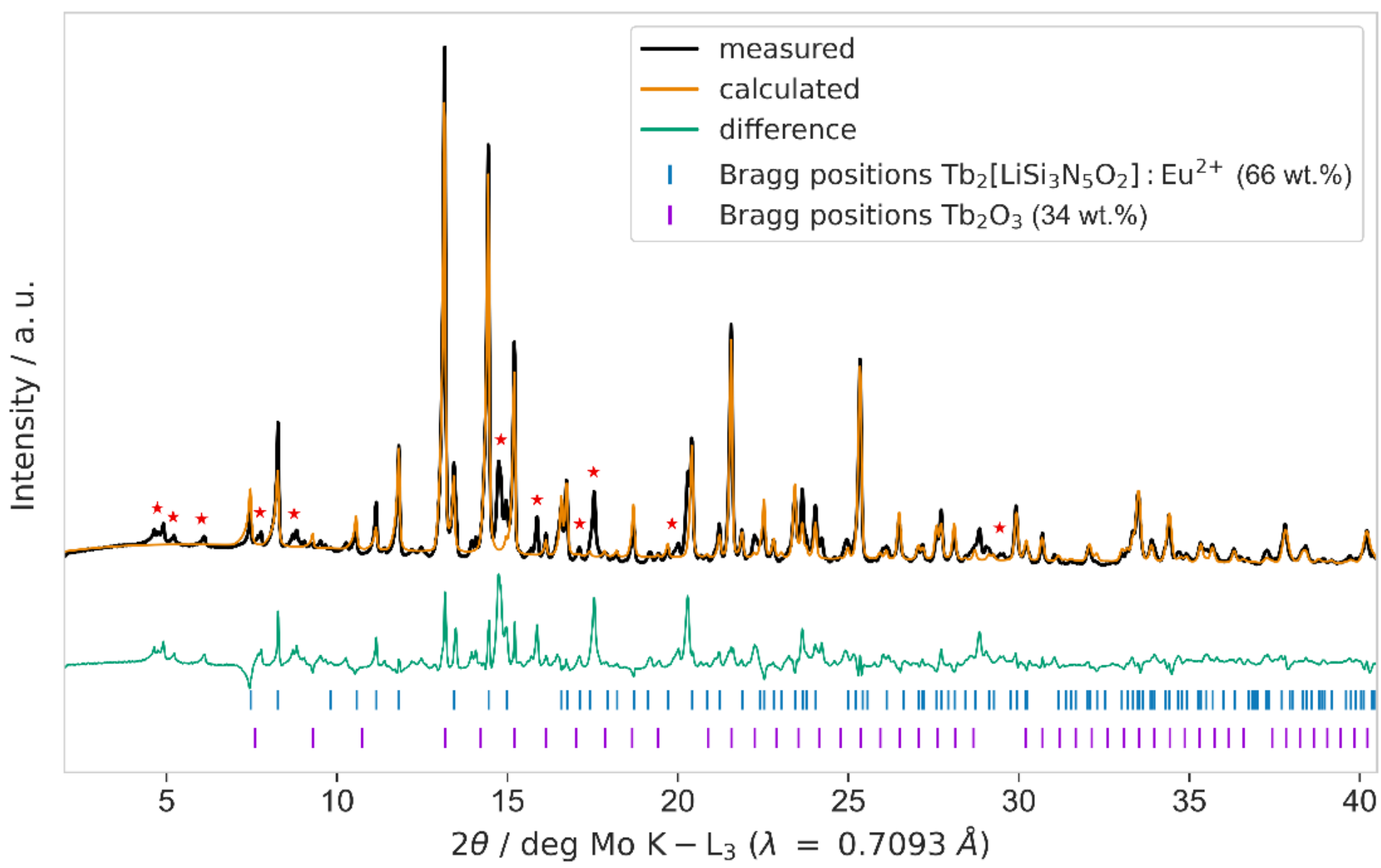

Figure S3: Rietveld fit of the measured powder diffraction data (black line) of $Tb_2[LiSi_3N_5O_2]$ activated with Eu^{2+}, the calculated pattern based on the theoretical reflections of the phases (orange line), and the difference plot (green line). Tick marks represent the theoretical reflection position of $Tb_2[LiSi_3N_5O_2]$ (blue tick marks; 66 wt.%) and of the secondary phase Tb_2O_3 (purple tick marks; 34 wt.%). Reflections that are not identified are asterisked (red).

Table S8: Results of the Rietveld refinement of the powder samples for $RE_2[LiSi_3N_5O_2]$ (RE = Ce, Nd, Sm) and $RE_2[LiSi_3N_5O_2]:Eu^{2+}$ (RE = Gd[1], Tb).

**Composition** of $RE_2[LiSi_3N_5O_2]$ with RE	**Ce**	**Nd**	**Sm**	**Gd**[1]	**Tb**
$RE_2[LiSi_3N_5O_2]$ / wt.%	49	53	61	90	66
Other phases / wt.%	LiF 51	LiF 21 / $NdSiNO_2$ 19 / NdOF 7	LiF 32 / SmOF 7	Gd_2O_3 10	Tb_2O_3 34 / n.d.
**Crystallographic data**					
Diffractometer	STOE STADI P				
Radiation	Mo K-L3 (λ = 0.7093 Å)				
a / Å	7.8340(2)	7.7760(3)	7.7495(3)	7.7248(2)	7.7106(6)
c / Å	5.0250(3)	4.9937(3)	4.9583(2)	4.9300(2)	4.9218(6)
V / Å^3	308.39(3)	301.95(3)	297.77(2)	294.18(2)	292.62(6)
2θ-range / °	2.02 – 40.09	2.02 – 40.09	2.02 – 40.09	2.02 - 43.30	2.02 – 70.09
Θ-step size / °	0.015				
R_{exp} / %	1.37	1.42	3.81	13.23	1.60
R_{wp} / %	20.14	12.32	11.00	16.22	25.91
R_p / %	13.24	9.17	7.60	11.77	18.60
R_{Bragg} / %	6.393	5.076	3.453	3.928	10.352

Table S9: Wyckoff-positions, atomic coordinates, and isotropic displacement parameter for $Sm_2[LiSi_3N_5O_2]$ based on Rietveld refinement. Atomic coordinates were fixed to the values of the isotypic gadolinium compound during the refinement.

**Atom**	**Wyckoff-position**	***x***	***y***	***z***	B_{iso} / Å^2
Sm1	4*e*	0.16055	*x*+½	0.5034092	0.8187824
Si1	4*e*	0.64405	*x*+½	0.935643	0.5921763
Si2	2*a*	0	0	0	0.5211151
O1	4*e*	0.6423	*x*+½	0.2683231	0.7658813
N1	8*f*	0.0908	0.157159	0.2167274	0.7185072
N2	2*c*	0	½	0.1831	0.6553417
Li1	2*b*	0	0	½	1.894964

Table S10: Wyckoff-positions, atomic coordinates, and isotropic displacement parameter for $Ce_2[LiSi_3N_5O_2]$ based on Rietveld refinement. Atomic coordinates were fixed to the values of the isotypic neodymium compound during the refinement.

Atom	Wyckoff-position	*x*	*y*	*z*	B_{iso} / Å^2
Ce1	4*e*	0.1627879	*x*+½	0.5106672	0.634813
Si1	4*e*	0.64379	*x*+½	0.93944	0.4824263
Si2	2*a*	0	0	0	0.3860989
O1	4*e*	0.6426	*x*+½	0.273	0.7500899
N1	8*f*	0.0804	0.161	0.2058	0.5842806
N2	2*c*	0	½	0.1766	0.4974281
Li1	2*b*	0	0	½	1.642302

Table S11: Calculated bond valences using BLBS (∑V) and CHARDI-2015 (∑Q) for $Pr_2[LiSi_3N_5O_2]$ based on single-crystal data.[3–5]

Atom	Pr1	Li1	Si1	Si2	N1	N2	O1
**∑V**	2.76	0.97	3.82	3.78	-2.75	-3.20	-1.82
**∑Q**	2.97	1.05	3.92	4.18	-2.97	-3.05	-2.03

Table S12: Calculated bond valences using BLBS (∑V) and CHARDI-2015 (∑Q) for $Tb_2[LiSi_3N_5O_2]$ based on single-crystal data.[3–5]

Atom	Tb1	Li1	Si1	Si2	N1	N2	O1
**∑V**	3.27	0.98	4.29	3.53	-3.01	-3.33	-2.05
**∑Q**	2.97	1.05	3.91	4.19	-2.97	-3.06	-2.03

Table S13: Partial MAPLE values and MAPLE sums in kJ mol^{-1} for $RE_2[LiSi_3N_5O_2]$ (*RE* = Pr, Nd, Gd, Tb) based on single-crystal data.[6–10]

	$LiPr_2Si_3N_5O_2$	$LiNd_2Si_3N_5O_2$	$LiGd_2Si_3N_5O_2$	$LiTb_2Si_3N_5O_2$	References
RE^{3+}	4219.34	4236.20	4329.38	4367.77	3500-5100
**Li1^{+}**	829.28	829.28	852.48	775.00	550–860
**Si1^{4+}**	9737.32	9753.44	9783.72	9637.66	9000–10200
**Si2^{4+}**	9971.00	10000.32	9979.89	10058.16	9000–10200
**N1^{3-}**	5348.22	5364.74	5422.47	5459.47	4300–6200
**N2^{3-}**	5633.80	5650.59	5694.42	5577.20	4300–6200
**O1^{2-}**	2423.51	2431.78	2432.98	2479.80	2000–2800
**∑**	70576.52	70781.98	71308.85	71639.20	
M	84.76	85.01	85.64	86.03	

Table S14: Selected interatomic distances for $Nd_2[LiSi_3N_5O_2]$ in Å determined by single-crystal data (standard deviations in parentheses).

Atom 1	Atom 2	Distance	Count	Average value	Distance
Nd1	- O1	2.619(2)	×2		
	- O1	2.457(3)		**Ø Nd1-O**	**2.565(3)**
	- N1	2.492(2)	×2		
	- N1	2.767(2)	×2		
	- N2	2.401(3)		**Ø Nd1-N**	**2.584(3)**
Si1	- 01	1.664(3)		**Ø Si1-O**	**1.664(3)**
	- N1	1.759(3)	×2		
	- N2	1.690(2)		**Ø Si1-N**	**1.736(3)**
Si2	- N1	1.739(2)	×4	**Ø Si2-N**	**1.739(2)**
Li	- N1	2.030(2)	×4	**Ø Li1-N**	**2.030(2)**

Table S15: Fitting coefficients A, B, C for the quadratic fit for the temperature-dependent lattice parameters in $Gd_2[LiSi_3N_5O_2]$.

	A	B	C
$\boldsymbol{a}$	$1.9777 \cdot 10^{-8}$	$4.1396 \cdot 10^{-5}$	7.7245
$\boldsymbol{c}$	$8.2571 \cdot 10^{-9}$	$3.3374 \cdot 10^{-5}$	4.9298
$\boldsymbol{V}$	$2.0388 \cdot 10^{-6}$	$5.1470 \cdot 10^{-3}$	294.1476

Table S16: Cell parameters *a* and *c*, unit-cell volume *V*, polyhedral volume of the *RE* cation $V_{Poly.}$ and the average *RE*–*X* distance (<$\bar{d}_{RE-X}$>) for the compounds $RE_2[LiSi_3N_5O_2]$ (*RE* = Ce, Pr, Nd, Sm, Gd, Tb) (standard deviations in parentheses). The values are determined by Rietveld refinement and single-crystal data (asterisked). Abbreviation: r = ionic radius.[11]

RE	r / pm	V / Å³	a / Å	c / Å	$V_{Poly.}$ / Å³	<$\bar{d}_{RE-X}$> / Å
**Ce**	114	308.41(3)	7.8342(4)	5.0250(3)	29.60(3)	2.5925(2)
**Nd**	111	301.95(2)	7.7761(2)	4.9935(2)	29.00(2)	2.5725(2)
**Sm**	108	297.77(2)	7.7495(3)	4.9583(2)	28.19(3)	2.5503(1)
**Gd**	105	294.18(2)	7.7247(2)	4.9300(2)	27.86(2)	2.5407(2)
**Tb**	104	292.62(6)	7.7106(6)	4.9218(6)	27.71(6)	2.5362(2)
**Pr***	113	306.74(4)	7.8272(3)	5.0068(3)	29.46(4)	2.587(4)
**Nd***	111	301.95(2)	7.7761(2)	4.9935(2)	29.14(2)	2.577(4)
**Gd***	105	294.18(2)	7.7247(2)	4.9300(2)	27.82(2)	2.539(4)
**Tb***	104	291.8(3)	7.703(3)	4.918(3)	27.6(3)	2.54(5)

Table S17: Selected interatomic distances <d> and layer spacings <$d_{A\text{-}B}$> for the compounds $RE_2[LiSi_3N_5O_2]$ (*RE* = Ce, Pr, Nd, Sm, Gd, Tb) (standard deviations in parentheses). The values are determined by Rietveld refinement and single-crystal data (asterisked).

RE	**Ce**	**Pr***	**Nd**	**Sm**	**Gd**	**Tb**
<$\bar{d}_{Si\text{-}N}$> / Å	1.7485(1)	1.745(3)	1.7370(1)	1.7406(1)	1.7338(1)	1.7307(1)
<$\bar{d}_{Si\text{-}(O,N)}$> / Å	1.7261(2)	1.721(4)	1.7127(1)	1.7155(1)	1.7077(1)	1.7047(2)
<$\bar{d}_{Li\text{-}N}$> / Å	2.0428(2)	2.043(3)	2.0296(1)	2.0076(1)	2.0000(1)	1.9965(2)
<$d_{A\text{-}B}$> / Å	2.5125(2)	2.5034(1)	2.4955(2)	2.4792(2)	2.465(1)	2.4609(3)

Table S18: Selected interatomic angles of the tetrahedra linkage in the anionic substructure of the compounds $RE_2[LiSi_3N_5O_2]$ (*RE* = Ce, Pr, Nd, Sm, Gd, Tb). The values are determined by Rietveld refinement and single-crystal data (asterisked).

RE	Ce	Pr*	Nd	Sm	Gd	Tb
**Si2–N[2]–Si1**	118.79(1)°	119.1(2)°	118.70(1)°	118.04(1)°	118.25(1)°	118.24(1)°
**Si1–N[2]–Si1**	139.79(1)°	139.8(3)°	139.65(1)°	138.16(1)°	138.48(1)°	138.47(1)°

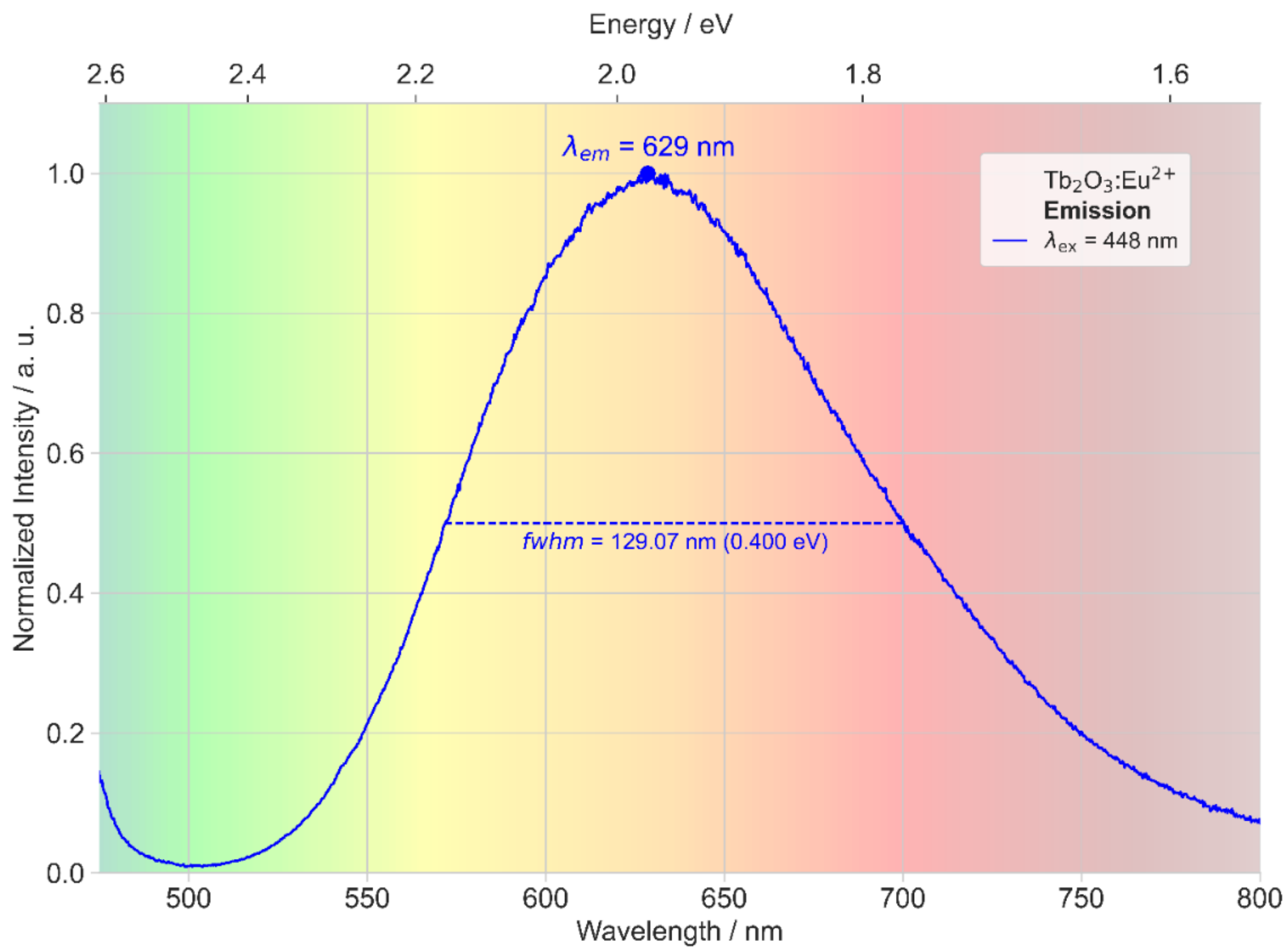

Figure S4: Emission spectrum of a single grain of $Tb_2O_3:Eu^{2+}$ upon excitation with blue light (λ_{exc} = 448 nm). The emission spectrum was taken over two seconds and averaged over five scans.

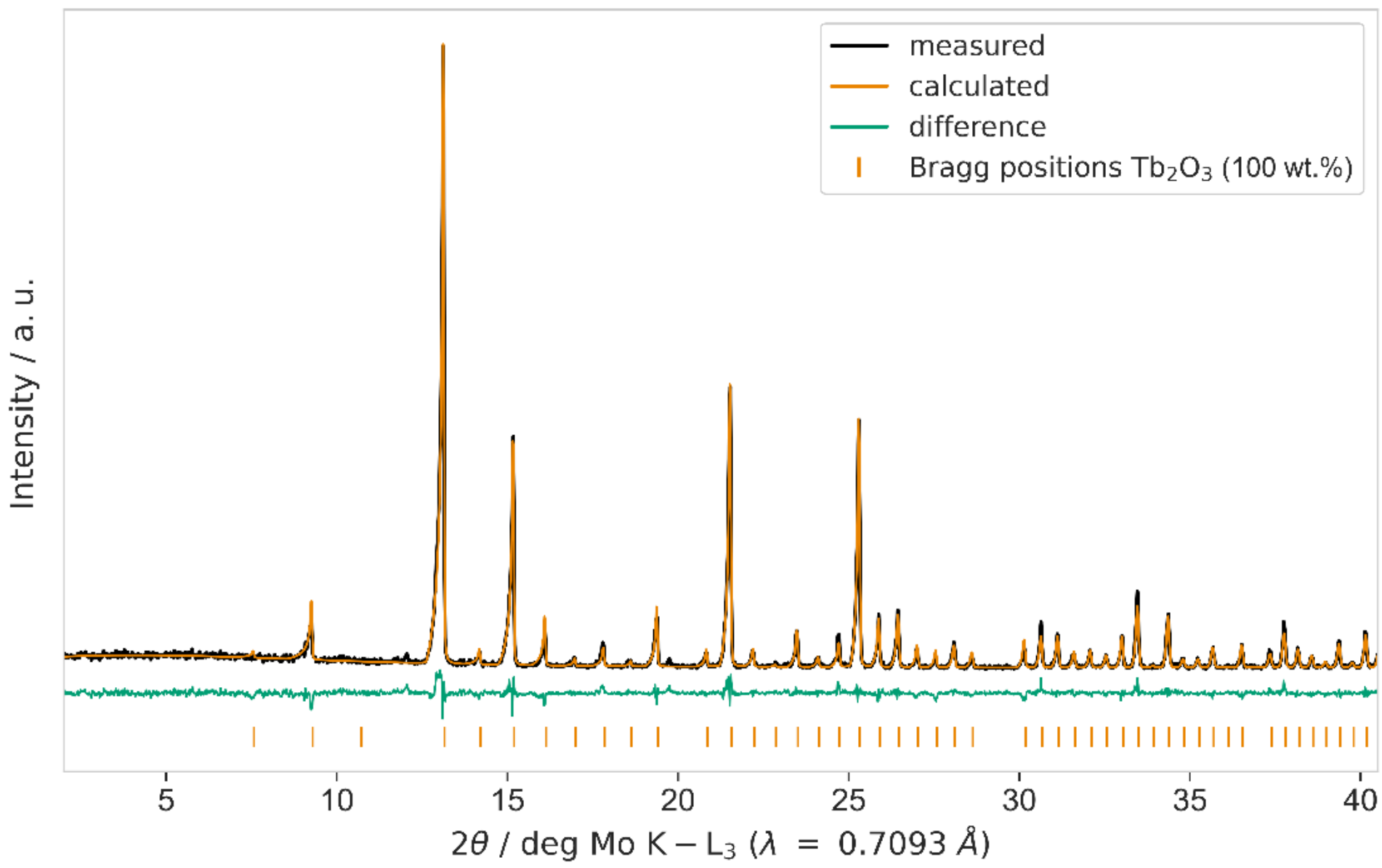

Figure S5: Rietveld fit of the measured powder diffraction data (black line) of $Tb_2O_3:Eu^{2+}$, the calculated pattern based on the theoretical reflections of Tb_2O_3 (orange line), and the difference plot (green line). Tick marks represent the theoretical reflection position of Tb_2O_3 (orange tick marks). High-temperature solid-state reactions in arc-welded tantalum tubes under an argon atmosphere were employed to obtain Eu^{2+}-doped Tb_2O_3. Therefore, the reactants Tb_2O_3 (97.44 mg, Alfa Aesar, >99.9 %) and EuF_2 (2.56 mg, Alfa Aesar, 99.9 %) were weighed in. The subsequent synthesis procedure was carried out according to the general procedure described in the experimental section.

References

1 M. N. van Os, K. M. Rießbeck, M. Seibald, C. Stoll, H. Huppertz *Solid State Sci.* 2026, **175**, 108240.
1 L. Krause, R. Herbst-Irmer, G. M. Sheldrick, D. Stalke *J. Appl. Cryst*. 2015, **48**, 3–10.
3 I. D. Brown, D. Altermatt *Acta. Cryst. B.* 1985, **41**, 244–247.
4 N. E. Brese, M. O'Keeffe *Acta. Cryst. B.* 1991, **47**, 192–197.
5 M. Nespolo, B. Guillot *J. Appl. Cryst.* 2016, **49**, 317–321.
6 R. Hoppe *Angew. Chem.* 1970, **82**, 7–16.
7 R. Hoppe *Angew. Chem.* 1966, **78**, 52–63.
8 P. Pust, W. Schnick *Z. Anorg. Allg. Chem.* 2012, **638**, 352–356.
9 K. Horky, W. Schnick *Eur. J. Inorg. Chem.* 2017, **2017**, 1107–1112.
10 M. Zeuner, S. Pagano, W. Schnick *Angew. Chem. Int. Ed.* 2011, **50**, 7754–7775.
11 R. D. Shannon *Acta. Cryst. A.* 1976, **32**, 751–767.
